# Medical Images Encryption Based on Adaptive-Robust Multi-Mode Synchronization of Chen Hyper-Chaotic Systems

**DOI:** 10.3390/s21113925

**Published:** 2021-06-07

**Authors:** Ali Akbar Kekha Javan, Mahboobeh Jafari, Afshin Shoeibi, Assef Zare, Marjane Khodatars, Navid Ghassemi, Roohallah Alizadehsani, Juan Manuel Gorriz

**Affiliations:** 1 Faculty of Electrical Engineering, Zabol Branch, Islamic Azad University, Zabol 1939598616, Iran; kikha_akbar@yahoo.com; 2 Electrical and Computer Engineering Faculty, Semnan University, Semnan 3513119111, Iran; mahbube.jafari@yahoo.com; 3 Faculty of Electrical Engineering, Biomedical Data Acquisition Lab (BDAL), K. N. Toosi University of Technology, Tehran 1631714191, Iran; afshin.shoeibi@gmail.com; 4 Faculty of Electrical Engineering, Gonabad Branch, Islamic Azad University, Gonabad 6518115743, Iran; 5 Faculty of Engineering, Mashhad Branch, Islamic Azad University, Mashhad 91735413, Iran; khodatars1marjane@gmail.com; 6 Computer Engineering Department, Ferdowsi University of Mashhad, Mashhad 9177948974, Iran; ghaseminavid76@gmail.com; 7 Institute for Intelligent Systems Research and Innovation (IISRI), Deakin University, Waurn Ponds, VIC 3217, Australia; ralizadehsani@deakin.edu.au; 8 Department of Signal Theory, Networking and Communications, Universidad de Granada, 52005 Granada, Spain; gorriz@ugr.es

**Keywords:** medical images, encryption, multi-mode synchronization, robust control, adaptive control, time varying, lyapunov stability

## Abstract

In this paper, a novel medical image encryption method based on multi-mode synchronization of hyper-chaotic systems is presented. The synchronization of hyper-chaotic systems is of great significance in secure communication tasks such as encryption of images. Multi-mode synchronization is a novel and highly complex issue, especially if there is uncertainty and disturbance. In this work, an adaptive-robust controller is designed for multimode synchronized chaotic systems with variable and unknown parameters, despite the bounded disturbance and uncertainty with a known function in two modes. In the first case, it is a main system with some response systems, and in the second case, it is a circular synchronization. Using theorems it is proved that the two synchronization methods are equivalent. Our results show that, we are able to obtain the convergence of synchronization error and parameter estimation error to zero using Lyapunov’s method. The new laws to update time-varying parameters, estimating disturbance and uncertainty bounds are proposed such that stability of system is guaranteed. To assess the performance of the proposed synchronization method, various statistical analyzes were carried out on the encrypted medical images and standard benchmark images. The results show effective performance of the proposed synchronization technique in the medical images encryption for telemedicine application.

## 1. Introduction

Recently, telemedicine systems have been introduced to assist the diagnosis and treatment of various diseases [1,2,3]. These systems, through telecommunication systems and clinical data registration devices, have been able to yield good medical services between specialists and patients easily; the significance of this issue arises when some patients for any reason cannot attend medical specialty centers [4,5]. Specialist physicians can apply telemedicine to diagnose the disease using clinical data such as medical images and signals.

When sending and receiving clinical data in telecommunication channels, information security is one of the main dilemmas of telemedicine systems [6]; because telecommunication channels are not secure enough to send medical information. Various medical information of patients is confidential, and at the time of sending and receiving from the patient to the specialist must have a sufficient security communication channel and cannot be accessed by other individuals and organizations [7,8]. In order to enhance the security of patients’ information at the time of sending, various approaches are exploited that the most important of which are cryptographic techniques.

One of the most important fields of cryptography is chaotic systems [9,10]. The most significant feature of chaotic systems is the high reaction to small changes in the initial conditions [11,12]. Much research has been conducted in this field for the last two decades since Correll et al. [13,14,15] introduced the synchronization of chaotic systems. In recent years, the employment of chaotic systems in clinical data encryption has grown remarkably [16]. Because chaos systems mainly guarantee medical information confidentiality when sending and receiving from telecommunication channels [16].

Data encryption is usually done by chaotic or hyper-chaotic methods [17,18,19,20,21,22,23,24,25,26,27,28,29,30,31,32,33,34,35,36,37]. In some researches, chaotic or hyper-chaotic systems are used to encrypt peripheral data [38,39,40,41,42,43]. In recent years, researchers have focused on the encryption of medical data using chaotic methods [17,18,19,20,21,22,23,24,25,26,27,28,29,30,31,32,33,34,35]. Medical data contains important information about patients [41,44,45,46]. Therefore, the confidentiality of medical information is essential when sending it through telecommunication channels. Chaotic theory, as a nonlinear and complex phenomenon, plays an important role in increasing the security of medical data. In the following, a number of conducted investigations using cryptographic procedures in clinical data have been examined.

Lin et al. [17] used chaos theory to the encryption of electroencephalogram (EEG) signals. In this paper, the chaos-based EEG encryption system involves three levels of encryption and is implemented in the C# programming language.

A chaotic theory-based cryptographic method for clinical signals is presented in [18]. The proposed encryption algorithm is based on a logistic map with double chaotic layer encryption (DCLE). In this study, EEG, electrocardiograms (ECG), and blood pressure (BP) data from the PhysioBank database were acquired.

The idea of the optical chaos method for secure EEG signals transmission in telemedicine applications has been discussed in Shahzadi et al.’s research [19]. In the proposed scheme, a semiconductor laser source is utilized to generate optical chaos and conceals the EEG signal in the chaotic signal before transmission over the optical fiber. The generated data is then sent over optical fiber.

Applying chaotic modulation in intrinsic mode functions to encrypt medical ECG and EEG data is done in [20]. The results prove that the proposed method for encrypting and decrypting EEG and ECG signals containing chaotic behavior has satisfactory results.

In the research of Ibrahim et al. [21], medical image encryption was conducted using dynamic S-boxes and chaotic maps. Experimental results explicate that the proposed scheme has successfully passed all security tests in medical image encryption.

Gafsi et al. [22] proposed an improved chaotic system for medical image encryption. In this paper, a complex chaotic-based pseudorandom number generator (PRNG) has been designed to generate a high-quality encryption key. The generated key indicates the high complexity behavior of the approach.

In [23], a medical image encryption method has been proposed based on a chaotic map and fractional discrete cosine transform (FrDCT) coefficients. The proposed method comprises two steps of applying FrDCT on an image and, after that, a chaotic map on the coefficients.

The fourth-order chaotic system for clinical data encryption introduced in reference [24]. They calculated a variety of significant statistical parameters, such as correlation for the encrypted images in the proposed scheme.

An encryption algorithm based on the properties of block encryption, 4-dimensional logistics map and, DNA systems has been introduced by Stalin et al. [25]. In this method, multiple key sequences are generated first. In the second step, the pixel is substituted by a 4D nonlinear logistic map. Finally, encryption is performed using DNA rules to ensure that the various blocks are securely encrypted.

A chaotic system based on the Arnold cat method for medical images encryption in reference [26] is presented. The results demonstrate the high security and robustness of the proposed approach for secure medical image transmission.

In another research, Choi et al. [27] presented a secure and robust medical images encryption method. The proposed method include two permutations based on plain image and Latin square (PPILS) and bi-directional adaptive diffusion.

Choi et al. [28] proposed the NCA-based color medical image encryption algorithm and the generalized 3D chaotic cat map in another study. In this work, the NCA is an effective PRNG generating nonlinear pseudorandom sequences. Generalized 3D chaotic cat mapping is also adopted to create effective shuffle in encryption of color medical images.

A medical image encryption system based on multiple chaotic and message-digest algorithm 5 (MD5) has been discussed in [29]. The system employs two rounds of chaotic diffusion using XOR bit operation to encrypt an image. The results show that the proposed method is highly efficient in medical image encryption.

Designing a cryptographic algorithm based on pseudorandom sequence generation using a generalized double humped logistic map was done in [30], which ensures the transmission of magnetic resonance imaging (MRI) and X-ray images with high security. In this work, various statistical analyzes have been exploited.

Chaotic map-based authentication for telecare medicine information systems (TMISs) was examined by Li et al. in [31]. The results show that the proposed method presented in this paper can be applied in e-health services.

A method based on the integration of fused coupled chaotic map (FCCM) to encrypt ECG signals has been proposed in Pandey et al.’s study [32]. The FCCM generates four different chaotic maps based on control parameters. The effective-ness of the proposed approach has been confirmed using various analyzes, including key sensitivity, key space, correlation evaluation, and histogram analysis.

In the research of Sangavi et al. [33], the method (MMIE) using Rossler dynamical system and sine map (Sine) for medical image encryption has been proposed. For the method, statistical metrics such as histogram analysis, entropy, differential, and correlation have been computed.

Presented by researchers in [34] is an improved technique for medical image encryption based on discrete wavelet transform (DWT), discrete cosine transform (DCT) and, singular value decomposition (SVD) transformations. Additionally, the security of the method has been improved by exploiting 2D logistic maps based on chaotic cryptography.

In Zheng et al.’s [35] study, the data encryption standard (DES) and elliptic curves cryptography (ECC) algorithms are used for EEG signals. The results reveal that the ECC method has superior performance in encryption and decryption of EEG signals.

In this paper, a novel medical images encryption based on synchronization of hyper chaotic systems is presented. The main purpose of providing synchronization methods is to ad-just the controller parameters in chaotic systems properly. In order to synchronize, various control methods such as adaptive control [47,48], sliding mode [49,50], fuzzy [51,52], optimal [53,54], predictive [55,56], etc. have been proposed so far. Chaotic synchronization is such that the state paths of the following system follow the state paths of a base system [57,58].

Due to the numerous applications of synchronization, various other methods have been proposed that have different applications based on the existence of chaos and uncertainty in them. The following are some of the newest synchronization methods. In [59], the idea of synchronization based on the sliding mode control method and developing the advanced encryption standard algorithm is presented. Synchronization of chaotic systems based on additional Fourier modes is one of the newest methods proposed by [60]. Ding et al. [38] performed the encryption based on fractional-order Henon chaotic map and the discrete-time wavelet transform for a hyper-chaotic system. A new encryption idea based on the discrete-time sliding mode method and the improved El-Gamal encryption system is presented in [61]. Synchronization with the optimized proportional integral derivative (PID) controller with particle swarm optimization (PSO) algorithm is another new method in this field that has been introduced by [62].

In this paper, the main idea of medical image encryption is based on robust adaptive control. Also, the synchronization method is multi-mode. The multi-state synchronization with the presence of uncertainty, disturbance, unknowns, and variable parameters has been carried out. The proposed synchronization method is used alongside the Chen hyper chaotic system to encrypt medical images. In the provided example, one Chen hyper-chaotic system is considered as the master system, and two Chen hyper chaotic systems are considered as the slave systems. According to the simulations, the robustness of the proposed method and the convergence of all error types to zero verify its capabilities. In the following, taking the advantage of masking technique, it is possible to use the proposed synchronization method for medical images encryption. For encryption, first, a variety of standard benchmark images have been employed. Then, the chest CT and X-ray images of COVID-19 patients with various noises have been examined. Also, to accurately verify the performance of the proposed method, a variety of important cryptographic parameters including histogram analysis, correlation, differential attack, PSNR and information entropy of CT images with different noises have been calculated to represent the performance of the proposed method correctly. The results reveal that the synchronization technique of the proposed hyper-chaotic system for encrypting standard images and medical images has shown successful results and can therefore be adopted as an encryption section in a COVID-19 detection telemedicine system. The proposed approach is capable of encrypting other medical images.

In the following, other sections of the paper are presented. The second part is devoted to the theories of the proposed synchronization method. In the Section 3, the idea of encrypting medical images and standard benchmarks based on the chaotic masking method is expressed. The implementation of the proposed synchronization method on the Chen hyper-chaotic system is introduced in Section 4. The types of statistical parameters to show the efficiency of the proposed synchronization method in the encryption of different images are provided in the Section 5. The Section 6 is devoted to the results of the proposed method in encrypting medical images and standard benchmarks. The advantages and disadvantages of the proposed method are described in Section 7. Finally, the discussion and conclusion are presented in Section 8.

## 2. Formulation of the Theorem

This section first describes the problem of synchronization of multi-mode transitive chaotic systems. The multiple loop synchronization is then checked. In both cases, the adaptive laws and controllers are designed using the adaptive control method.

### 2.1. **Comparative Synchronization between More Response Systems and a Master System**

**Figure 1:**
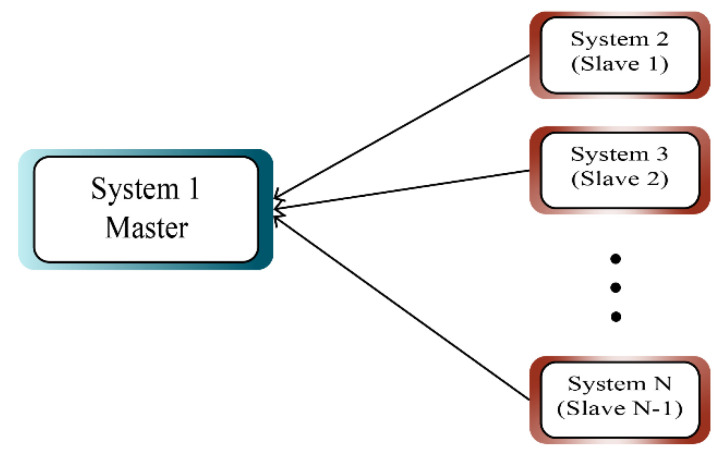
Structure of transmission synchronization.

Figure 1 shows the synchronization model between a master system and several slave systems.

The master chaotic system is defined as follows [48]:

(1) $${\dot{x}}_{1}=f_{1}\left(x_{1}\right)+H_{1}\left(x_{1}\right)\theta_{1}\left(t\right)$$

where $x_{1}\left(t\right)={\left[x_{11},x_{12},\cdots ,x_{1n}\right]}^{T}$ is the state vector of the system. $f_{1}(x_{1}\left(t\right))={\left[f_{11},f_{12},\cdots ,f_{1n}\right]}^{T}$ is the continuous function, $H_{1}(x_{1}\left(t\right))={\left[H_{11},H_{12},\cdots ,H_{1n}\right]}^{T}$ is matrix function and $\theta_{1}\left(t\right)={\left[\theta_{11},\theta_{12},\cdots ,\theta_{1n}\right]}^{T}$ is the main parameters of the master system, which are piecewise constant.

There are (*N* − 1) chaotic slave systems with control function as given by (2) [63]:

(2) $${\dot{x}}_{i}=f_{i}\left(x_{i}\right)+H_{i}\left(x_{i}\right)\theta_{i}\left(t\right)\;i=2,\;3,\;\cdots ,\;N$$

where $x_{i}\left(t\right)={\left[x_{i1},x_{i2},\cdots ,x_{in}\right]}^{T}$ is the state vector of the *i*th system, $f_{i}(x_{1}\left(t\right))={\left[f_{i1},f_{i2},\cdots ,f_{in}\right]}^{T}\;$is the continuous function, $H_{i}(x_{i}\left(t\right))={\left[H_{i1},H_{i2},\cdots ,H_{in}\right]}^{T}$ is matrix function, $\theta_{i}\left(t\right)={\left[\theta_{i1},\theta_{i2},\cdots ,\theta_{in}\right]}^{T}$ are the main parameters which are piecewise constant of the *i*th slave system, and $u_{i-1}\left(t\right)={\left[u_{i-1.1}\left(t\right),u_{i-1.2}\left(t\right),\cdots ,u_{i-1.n}\left(t\right)\right]}^{T}$, is the control function of *i*th system. Therefore, according to Equations (1) and (2), synchronization of the chaotic system with the control function is as follows:

(3) $$\begin{array}{c} \dot{x_{1}}=f_{1}\left(x_{1}\right)+H_{1}\left(x_{1}\right)\theta_{1}\left(t\right)\\ \dot{x_{2}}=f_{2}\left(x_{2}\right)+H_{2}\left(x_{2}\right)\theta_{2}\left(t\right)+u_{1}\left(t\right)\\ \vdots \\ \dot{x_{N}}=f_{N}\left(x_{N}\right)+H_{N}\left(x_{N}\right)\theta_{N}\left(t\right)+u_{N-1}\left(t\right) \end{array}$$

In the form of multiple synchronization mode, the synchronization error is defined as follows:

$$e_{i-1}\left(t\right)=x_{i}\left(t\right)-x_{1}\left(t\right)\;i=2,3,\cdots ,N\;$$

**Definition** **1.** *Chaotic system (3) using the controllers* $u_{i}\left(t\right)$ *and unknown parameters have multi-state transformative synchronization if the following condition is held:*

$$\underset{t\to \infty }{\lim}||e_{i-1}\left(t\right)||=\underset{t\to \infty }{lim}\;||x_{i}\left(t\right)-x_{1}\left(t\right)||\;\to 0\;i=2,3,\cdots ,N\;$$

*where the error dynamic defined as follows:*

(4) $${\dot{e}}_{i-1}\left(t\right)=f_{i}\left(x_{i}\right)-f_{1}\left(x_{1}\right)+H_{i}\left(x_{i}\right)\theta_{i}\left(t\right)-H_{1}\left(x_{1}\right)\theta_{1}\left(t\right)+u_{i-1}\;i=2,3,\;\cdots ,N-1$$

*is met, then the adaptive transmission synchronization between N chaotic systems with unknown parameters is realized.*

*The design of controllers and adaptive laws to achieve the above goal is based on Lyapunov’s function, and the synchronization under the mode of transmission synchronization is performed. The control law for* $u_{1}\left(t\right),u_{2}\left(t\right),\ldots ,u_{N-1}\left(t\right)$ *can be designed as follows:*

(5) $$u_{i-1}\left(t\right)=-f_{i}\left(x_{i}\right)+f_{1}\left(x_{1}\right)-H_{i}\left(x_{i}\right){\hat{\theta }}_{i}\left(t\right)+H_{1}\left(x_{1}\right){\hat{\theta }}_{1}\left(t\right)+K_{i-1}e_{i-1}\;\;i=2,3,\;\cdots ,N-1\;$$

*in which:*

$$K_{i-1}=K_{i-1}^{1}+K_{i-1}^{2},{\left(K_{i-1}^{1}\right)}^{T}=-K_{i-1}^{1},\;K_{i-1}^{2}=-diag\left(k_{i-1.1}^{.},\;k_{i-1.2}^{.},\cdots ,\;k_{i-1.n}^{.}\right),\;k_{i-1,j}^{.}>0,\;j=1,2,\cdots ,n$$

*Therefore, error dynamics are given as follows:*

(6) $${\dot{e}}_{i-1}\left(t\right)=H_{i}\left(x_{i}\right){\tilde{\theta }}_{i}\left(t\right)-H_{1}\left(x_{1}\right){\tilde{\theta }}_{1}\left(t\right)+K_{i-1}e_{i-1}\;i=2,3,\;\cdots ,N-1$$

*where* ${\hat{\theta }}_{i}\left(t\right)$ *is an approximation of* $\theta_{i}\left(t\right)$ *and* ${\tilde{\theta }}_{i}\left(t\right)=\theta_{i}\left(t\right)-{\hat{\theta }}_{i}\left(t\right)$ *is an approximation error.*

**Theorem** **1.** *Transmission synchronization of chaotic systems (3) with controls (5), error dynamics (6) and update laws (9a), (9b), (10a), and (10b) is guaranteed.*

**Proof.**  Consider the following Lyapunov function:

(7) $$V\left(e.\tilde{\theta }\right)=\frac{1}{2}(\overset{N}{\underset{i=2}{\sum }}[e_{i-1}^{T}e_{i-1}+{\tilde{\theta }}_{i}^{T}{\tilde{\theta }}_{i}]+{\tilde{\theta }}_{1}^{T}{\tilde{\theta }}_{1})$$

The derivative of Lyapunov’s function (7) is obtained according to Equation (8)

(8) $$\frac{d}{dt}V=\overset{N}{\underset{i=2}{\sum }}[e_{i-1}^{T}\left(H_{i}\left(x_{i}\right){\tilde{\theta }}_{i}-H_{1}\left(x_{1}\right){\tilde{\theta }}_{1}+\frac{1}{2}\left(K_{i-1}+K_{i-1}{}^{T}\right)e_{i-1}\right)+{\tilde{\theta }}_{i}^{T}{\dot{\tilde{\theta }}}_{i}]+{\tilde{\theta }}_{1}^{T}{\dot{\tilde{\theta }}}_{1}$$

Therefore, the parameters laws are chosen as follows:

(9a) $${\dot{\tilde{\theta }}}_{i}=-\left(H_{i}\left(x_{i}\right)e_{i-1}+\sigma_{i}{\tilde{\theta }}_{i}\right)\;\sigma_{i}>0\;i=2,3,\cdots ,N$$

(9b) $${\dot{\tilde{\theta }}}_{1}=\overset{N-1}{\underset{i=2}{\sum }}H_{1}\left(x_{1}\right)e_{i-1}-\sigma_{1}{\tilde{\theta }}_{1}\;\sigma_{1}>0$$

If $\theta_{i}$ are constant then ${\dot{\theta }}_{i}=0$ and the parameters estimation laws are determined as follows:

(10a) $${\dot{\hat{\theta }}}_{i}=-{\dot{\tilde{\theta }}}_{i}=H_{i}\left(x_{i}\right)e_{i-1}+\sigma_{i}{\tilde{\theta }}_{i}\;\sigma_{i}>0\;i=2,3,\cdots ,N\;$$

(10b) $${\dot{\hat{\theta }}}_{1}=-{\dot{\tilde{\theta }}}_{1}=-\overset{N-1}{\underset{i=2}{\sum }}H_{1}\left(x_{1}\right)e_{i-1}+\sigma_{1}{\tilde{\theta }}_{1}\;\sigma_{1}>0$$

By setting the estimation laws in (8), we will have:

(11) $$\frac{d}{dt}V=\overset{N}{\underset{i=2}{\sum }}[e_{i-1}^{T}K_{i-1}^{2}e_{i-1}-\sigma_{i}{\tilde{\theta }}_{i}^{T}{\tilde{\theta }}_{i}]-\sigma_{1}{\tilde{\theta }}_{1}^{T}{\tilde{\theta }}_{1}\;\leq -\varphi \;V$$

where $\varphi =\underset{i,j}{\min}\left(\sigma_{i},k_{i-1.j}^{.}\right)>0$ and $K_{i-1}^{2}\;$are Hurwitz. So system (6) is stable and $||e_{i}\left(t\right)||\to$0. Therefore, obtain an adaptive synchronization between (*N* − 1) slave systems and a master system. □

**Note** **1.** *If the systems are time varying:* $\theta_{i}=\theta_{i}\left(t\right)$ *Equations (9a) and (9b) are still stablished, then in these cases the Theorem 1 is also established. Since the synchronization error converges to zero, the Equations (10a) and (10b) are converted as follows for relatively long periods of time:*

$${\dot{\tilde{\theta }}}_{i}=-\sigma_{i}{\tilde{\theta }}_{i}\;\Rightarrow {\tilde{\theta }}_{i}\left(t\right)={\tilde{\theta }}_{i}\left(0\right)\exp\left(-\sigma_{i}t\right)\to 0\;\sigma_{i}>0\;\;i=1,2,3,\cdots ,N$$

*Therefore, if* $\theta_{i}\left(t\right)$ *is a vector with piecewise constant element and the appropriate time interval between changes in any element, the updating rule (10) is established and the parameters are estimated accurately. If* $\left|\frac{d}{dt}\theta_{i}\left(t\right)\right|<s_{i}<1$ *then the update Equation (10) are also well established.*

### 2.2. **Circular Synchronization of Multiple Chaotic Systems with Unknown Parameters**

**Figure 2:**
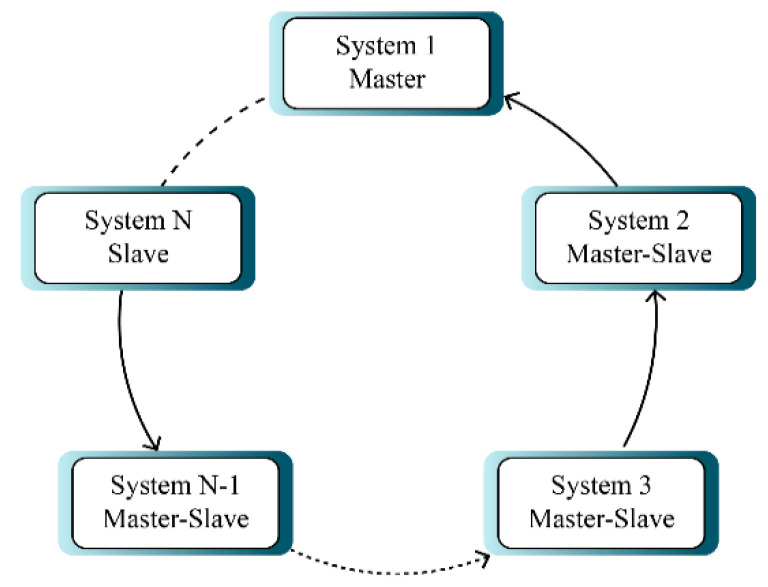
Structure of circular synchronization.

Figure 2 displays circular multi-mode synchronization. In this type of synchronization, systems 1 and *N* have the role of master and slave respectively and other systems both have the role of slave and master at the same time (*i*-th system is master role of *i* + 1-th system), which makes it more complex.

According to Figure 2, the systems are synchronized with a circular structure. In this case, the first chaotic system is described by the following expression:

(12) $${\dot{x}}_{1}\left(t\right)=f_{1}\left(x_{1}\right)+H_{1}\left(x_{1}\right)\theta_{1}\left(t\right)\;$$

The (*N* − 1) chaotic systems are as follows:

(13) $$\left\{ \begin{array}{c} {\dot{x}}_{1}\left(t\right)=f_{1}\left(x_{1}\right)+H_{1}\left(x_{1}\right)\theta_{1}\left(t\right)\\ {\dot{x}}_{2}\left(t\right)=f_{2}\left(x_{2}\right)+H_{2}\left(x_{2}\right)\theta_{2}\left(t\right)+m_{1}\left(t\right)\\ \vdots \\ {\dot{x}}_{N}\left(t\right)=f_{N}\left(x_{N}\right)+H_{N}\left(x_{N}\right)\theta_{N}\left(t\right)+m_{N-1}\left(t\right) \end{array}\right.$$

The control input is

$$m_{i-1}\left(t\right)={\left[m_{i-1.1},m_{i-1.2},\cdots ,m_{i-1.n}\right]}^{T}$$

For *N* chaotic system described by Equation (13), if there are adaptive controllers, so that the error dynamics systems are defined as follows:

(14) $$r_{i}\left(t\right)=x_{i+1}\left(t\right)-x_{i}\left(t\right)\;i=1,2,3,\cdots ,N-1\;{\dot{r}}_{i-1}\left(t\right)=f_{i}\left(x_{i}\right)-f_{i-1}\left(x_{i-1}\right)+H_{i}\left(x_{i}\right)\theta_{i}-H_{i-1}\left(x_{i-1}\right)\theta_{i-1}+m_{i-1}-m_{i-2}\;i=2,\;3,\;\cdots ,N-1$$

and condition:

$$\underset{t\to \infty }{\lim}||r_{i-1}\left(t\right)||=\underset{t\to \infty }{\lim}||x_{i}\left(t\right)-x_{i-1}\left(t\right)||\;\to 0\;i=2,3,\cdots ,N$$

is met then a circular synchronization between the *N* chaotic system and the unknown parameters is realized.

**Theorem** **2.** *The following statements are used to synchronize in both circular and transmission modes.*

*(A)*  *If there is a transmission and circular synchronization with the u(t) and m(t) controllers. Then:*
  $$\forall i,j\;:\;\underset{t\to \infty }{lim}||x_{i}\left(t\right)-x_{j}\left(t\right)||\;\to 0$$
*(B)*  *If transmission synchronization is established, circular synchronization is also realized and vice versa.*

**Proof.**  Assume that a transmission synchronization is established, so: $\forall \;i\;:\;e_{i}\left(t\right)\to 0$ so:

$$\forall \;i,j:||x_{i}\left(t\right)-x_{j}\left(t\right)||=||(x_{i}\left(t\right)-x_{1}\left(t\right))-(x_{j}\left(t\right)-x_{1}\left(t)\right)||\leq ||e_{i}\left(t\right)||+||e_{j}\left(t\right)||\to 0$$

If a circular synchronization is established (assuming i>j):

(15) $$\begin{array}{cc} \forall \;i,j:||x_{i}\left(t\right)-x_{j}\left(t\right)|| & \\ & =||(x_{i}\left(t\right)-x_{i-1}\left(t\right))+\left(x_{i-1}\left(t\right)-x_{i-2}\left(t\right)\right)+\cdots +(x_{j+1}\left(t\right)\\ & -x_{j}\left(t\right))||\leq ||r_{i-1}\left(t\right)||+\cdots +||r_{j}\left(t\right)||\leq \sum_{k=j}^{i-1}||r_{k}\left(t\right)||\to 0 \end{array}$$

 □

**Proof.**  The following relationships between errors are established in two synchronization modes:

$$r_{i}\left(t\right)\begin{array}{c} =\{\begin{array}{c} e_{i}\left(t\right)-e_{i-1}\left(t\right)\;i=2,3,\cdots ,N-1\;\\ e_{i}\left(t\right)\;i=1 \end{array},\;\\ \; \end{array}\;e_{i}\left(t\right)=\overset{i}{\underset{k=1}{\sum }}r_{k}\left(t\right)$$

If a transmission synchronization is established, $e_{i}\left(t\right)\to 0\;$therefore:

$$||r_{i}\left(t\right)||=\{\begin{array}{cc} ||e_{i}\left(t\right)-e_{i-1}\left(t\right)||\leq ||e_{i}\left(t\right)||+||e_{i-1}\left(t\right)||\to 0 & i\geq 2\\ ||e_{1}\left(t\right)||\to 0 & i=1 \end{array}$$

So circular synchronization is established. Conversely, suppose a circular synchronization is established, $r_{i}\left(t\right)\to 0$ therefore:

$$||e_{i}\left(t\right)||=||\overset{i}{\underset{k=1}{\sum }}r_{k}\left(t\right)||\leq \overset{i}{\underset{k=1}{\sum }}||r_{k}\left(t\right)||\to 0$$

Therefore, transmission synchronization is also established. So the two types of synchronization are equivalent. □

**Theorem** **3.** *The control law is same for transmission synchronization and circular synchronization. In other words:* $m_{i}\left(t\right)=u_{i}\left(t\right)$ *.*

**Proof.**  Using the relationship between two types of errors:

$${\dot{e}}_{i}\left(t\right)={\dot{e}}_{i-1}\left(t\right)+{\dot{r}}_{i}\left(t\right)$$

Which is obtained by placing in Equations (2) and (14):

(16) $$u_{i-1}\left(t\right)=u_{i}\left(t\right)-\left(m_{i}\left(t\right)-m_{i-1}\left(t\right)\right)\;i\geq 2$$

For *i* = 1, we have:

$$e_{1}\left(t\right)=r_{1}\left(t\right)\Rightarrow {\dot{e}}_{1}\left(t\right)={\dot{r}}_{1}\left(t\right)\Rightarrow m_{1}\left(t\right)=u_{1}\left(t\right)$$

By placing in Equation (16), we have: $m_{i}\left(t\right)=u_{i}\left(t\right)$ so the proof is completed. □

### 2.3. **Synchronization with the Presence of Disturbance and Uncertainty in the System**

In this case, the master and slave systems are accompanied by disturbance and uncertainty as follows:

(17) $$\left\{ \begin{array}{c} {\dot{x}}_{1}=f_{1}\left(x_{1}\right)+H_{1}\left(x_{1}\right)\theta_{1}\left(t\right)+\Delta f_{1}(x_{1})+D_{1}(t)\\ {\dot{x}}_{2}=f_{2}\left(x_{2}\right)+H_{2}\left(x_{2}\right)\theta_{2}\left(t\right)+\Delta f_{2}(x_{2})+D_{2}\left(t\right)+u_{1}\left(t\right)\\ \vdots \\ {\dot{x}}_{N}=f_{N}\left(x_{N}\right)+H_{N}\left(x_{N}\right)\theta_{N}\left(t\right)+\Delta f_{N}(x_{N})+D_{N}(t)+u_{N-1}\left(t\right) \end{array}\right.$$

where it is assumed that uncertainties and disturbances are bounded but with unknown bound.

$$\left|\Delta f_{i}\left(x_{i}\right)\right|\leq \gamma_{i}g_{i}(x_{i}),\;\left|D_{i}\left(t\right)\right|\;\leq d_{i}\;i=1,2,\ldots ,N$$

where $\gamma_{i}$ and *d_i_* are constant but indefinite and $g_{i}(x_{i})$ is a definite function and in the special case$\;g_{i}(x_{i})=\left|x_{i}\right|$. The dynamics of the errors are as follows:

(18) $${\dot{e}}_{i-1}\left(t\right)=f_{i}\left(x_{i}\right)-f_{1}\left(x_{1}\right)+H_{i}\left(x_{i}\right)\theta_{i}\left(t\right)-H_{1}\left(x_{1}\right)\theta_{1}\left(t\right)+\Delta f_{i}\left(x_{i}\right)-\Delta f_{1}\left(x_{1}\right)+D_{i}\left(t\right)\;-D_{1}\left(t\right)+u_{i-1}\left(t\right)\;i=2,3,\;\cdots ,N-1$$

By defining the control function as follows:

(19) $$u_{i-1}\left(t\right)=-f_{i}\left(x_{i}\right)+f_{1}\left(x_{1}\right)-H_{i}\left(x_{i}\right){\hat{\theta }}_{i}\left(t\right)+H_{1}\left(x_{1}\right){\hat{\theta }}_{1}\left(t\right)+K_{i-1}e_{i-1}+{\bar{u}}_{i-1}\left(t\right)\;i=2,3,\;\cdots ,N-1$$

where ${\hat{\theta }}_{i}\left(t\right)$ is an estimate of $\theta_{i}\left(t\right)$ and ${\bar{u}}_{i-1}\left(t\right)$ is part of the control function which is introduced below. By placing the control function in (18), the dynamics of errors are as follows:

(20) $${\dot{e}}_{i-1}\left(t\right)=H_{i}\left(x_{i}\right){\tilde{\theta }}_{i}\left(t\right)-H_{1}\left(x_{1}\right){\tilde{\theta }}_{1}\left(t\right)+\Delta f_{i}\left(x_{i}\right)-\Delta f_{1}\left(x_{1}\right)+D_{i}\left(t\right)-D_{1}\left(t\right)+K_{i-1}e_{i-1}+{\bar{u}}_{i-1}\left(t\right)\;\;i=2,3,\;\cdots ,N-1$$

**Theorem** **4.** *The dynamic system of error Equation (20) under control Equation (19), Equation (25) and update laws Equations (29) and (31) is stable and synchronization errors are convergent to zero despite uncertainty and disturbance.*

**Proof.**  by defining the Lyapunov function as follows:

(21) $$V=\frac{1}{2}(V_{e}+V_{\theta }+V_{\gamma }+V_{d})$$

where:

$$V_{e}=\overset{N}{\underset{i=2}{\sum }}e_{i-1}^{T}e_{i-1}\;V_{\theta }=\overset{N}{\underset{i=2}{\sum }}{\tilde{\theta }}_{i}^{T}{\tilde{\theta }}_{i}+{\tilde{\theta }}_{1}^{T}{\tilde{\theta }}_{1}.$$

$$V_{\gamma }=\overset{N}{\underset{i=2}{\sum }}{\tilde{\gamma }}_{i}{}^{2}+{\tilde{\gamma }}_{1}{}^{2}\;V_{d}=\overset{N}{\underset{i=2}{\sum }}{\tilde{d}}_{i}{}^{2}+{\tilde{d}}_{1}{}^{2}.$$

By calculating the derivative of Lyapunov’s function:

(22) $$\begin{array}{cc} \frac{d}{dt}V=\sum_{i=2}^{N}[e_{i-1}^{T} & (H_{i}\left(x_{i}\right){\tilde{\theta }}_{i}-H_{1}\left(x_{1}\right){\tilde{\theta }}_{1}+\Delta f_{i}\left(x_{i}\right)-\Delta f_{1}\left(x_{1}\right)+D_{i}\left(t\right)-D_{1}\left(t\right)\\ & +\frac{1}{2}\left(K_{i-1}+K_{i-1}{}^{T}\right)e_{i-1}+{\bar{u}}_{i-1}\left(t\right))+{\tilde{\theta }}_{i}^{T}{\dot{\tilde{\theta }}}_{i}+{\tilde{\gamma }}_{i}{\dot{\tilde{\gamma }}}_{i}+{\tilde{d}}_{i}{\dot{\tilde{d}}}_{i}]+{\tilde{\theta }}_{1}^{T}{\dot{\tilde{\theta }}}_{1}\\ & +{\tilde{\gamma }}_{1}{\dot{\tilde{\gamma }}}_{1}+{\tilde{d}}_{1}{\dot{\tilde{d}}}_{1}. \end{array}$$

Note that for ${\tilde{\theta }}_{i}\left(t\right)$ the same laws (9) and (10) are established, so Equation (22) becomes the following:

(23) $$\begin{array}{cc} \frac{d}{dt}V=\sum_{i=2}^{N}[e_{i-1}^{T} & \left(\Delta f_{i}\left(x_{i}\right)-\Delta f_{1}\left(x_{1}\right)+D_{i}\left(t\right)-D_{1}\left(t\right)+\frac{1}{2}(K_{i-1}+{K_{i-1}}^{T})e_{i-1}+{\bar{u}}_{i-1}(t)\right)\\ & +{\tilde{\gamma }}_{i}{\dot{\tilde{\gamma }}}_{i}+{\tilde{d}}_{i}{\dot{\tilde{d}}}_{i}]+{\tilde{d}}_{1}{\dot{\tilde{d}}}_{1}-\sum_{i=1}^{N}\sigma_{i}{\tilde{\theta }}_{i}^{T}{\tilde{\theta }}_{i} \end{array}$$

If $\Delta f_{i}{}^{j}\;,D_{i}{}^{j}\;,\;e_{i-1}^{j}\;$and ${\bar{u}}_{i-1}{}^{j}$ are the component j of the vectors$\;\Delta f_{i},\;D_{i},e_{i-1}$ and ${\bar{u}}_{i-1}\left(t\right)$ then:

(24) $$\frac{d}{dt}V=\overset{N}{\underset{i=2}{\sum }}\overset{n}{\underset{j=1}{\sum }}e_{i-1}^{j}\left(\Delta f_{i}{}^{j}-\Delta f_{1}{}^{j}+D_{i}{}^{j}-D_{1}{}^{j}+{\bar{u}}_{i-1}{}^{j}\right)+\overset{N}{\underset{i=2}{\sum }}\left({\tilde{\gamma }}_{i}{\dot{\tilde{\gamma }}}_{i}+{\tilde{d}}_{i}{\dot{\tilde{d}}}_{i}\right)\;+\overset{N}{\underset{i=2}{\sum }}e_{i-1}^{T}K_{i-1}^{2}e_{i-1}+{\tilde{\gamma }}_{1}{\dot{\tilde{\gamma }}}_{1}+{\tilde{d}}_{1}{\dot{\tilde{d}}}_{1}-\overset{N}{\underset{i=1}{\sum }}\sigma_{i}{\tilde{\theta }}_{i}^{T}{\tilde{\theta }}_{i}$$

Therefore:

(25) $$\frac{d}{dt}V\leq \overset{N}{\underset{i=2}{\sum }}\overset{n}{\underset{j=1}{\sum }}\left[\left|e_{i-1}^{j}\right|\left(\left|\Delta f_{i}{}^{j}\right|+\left|\Delta f_{1}{}^{j}\right|+\left|D_{i}{}^{j}\right|+\left|D_{1}{}^{j}\right|\right)+e_{i-1}^{j}{\bar{u}}_{i-1}{}^{j}\right]+\overset{N}{\underset{i=2}{\sum }}\left({\tilde{\gamma }}_{i}{\dot{\tilde{\gamma }}}_{i}+{\tilde{d}}_{i}{\dot{\tilde{d}}}_{i}\right)\;+\overset{N}{\underset{i=2}{\sum }}e_{i-1}^{T}K_{i-1}^{2}e_{i-1}+{\tilde{\gamma }}_{1}{\dot{\tilde{\gamma }}}_{1}+{\tilde{d}}_{1}{\dot{\tilde{d}}}_{1}-\overset{N}{\underset{i=1}{\sum }}\sigma_{i}{\tilde{\theta }}_{i}^{T}{\tilde{\theta }}_{i}$$

The bounded condition of disturbance and uncertainty can be developed on the components $\Delta f_{i}$ and $D_{i}$ as follows:

$$\left|\Delta f_{i}{}^{j}\right|\leq \underset{j}{\max}\left|\Delta f_{i}{}^{j}\right|\leq \left|\Delta f_{i}\left(x_{i}\right)\right|\leq \gamma_{i}g_{i}(x_{i})$$

$$\left|D_{i}{}^{j}\left(t\right)\right|\leq \underset{j}{\max}\left|D_{i}{}^{j}\left(t\right)\right|\leq \left|D_{i}\left(t\right)\right|\;\leq d_{i}$$

Which we have placed in the following equation.

(26) $$\frac{d}{dt}V\leq \overset{N}{\underset{i=2}{\sum }}\overset{n}{\underset{j=1}{\sum }}\left[\left|e_{i-1}^{j}\right|\left(\gamma_{i}\;g_{i}(x_{i})+\gamma_{1}\;g_{1}(x_{1})+d_{i}+d_{1}\right)+e_{i-1}^{j}{\bar{u}}_{i-1}{}^{j}\right]+\overset{N}{\underset{i=2}{\sum }}\left(\;{\tilde{\gamma }}_{i}{\dot{\tilde{\gamma }}}_{i}+{\tilde{d}}_{i}{\dot{\tilde{d}}}_{i}\right)\;+\overset{N}{\underset{i=2}{\sum }}e_{i-1}^{T}K_{i-1}^{2}e_{i-1}+{\tilde{\gamma }}_{1}{\dot{\tilde{\gamma }}}_{1}+{\tilde{d}}_{1}{\dot{\tilde{d}}}_{1}-\overset{N}{\underset{i=1}{\sum }}\sigma_{i}{\tilde{\theta }}_{i}^{T}{\tilde{\theta }}_{i}$$

If ${\bar{u}}_{i-1}{}^{j}\left(t\right)$ is defined as follows:

(27) $${\bar{u}}_{i-1}{}^{j}\left(t\right)=-\left({\hat{\gamma }}_{i}\;g_{i}(x_{i}\right)+{\hat{\gamma }}_{1}\;g_{1}(x_{1})+{\hat{d}}_{i}+{\hat{d}}_{1})\cdot \mathrm{sgn}\left(e_{i-1}^{j}\right)\;$$

Then:

(28) $$\frac{d}{dt}V\leq \overset{N}{\underset{i=2}{\sum }}\overset{n}{\underset{j=1}{\sum }}\left[\left|e_{i-1}^{j}\right|\left({\tilde{\gamma }}_{i}\;g_{i}(x_{i})+{\tilde{\gamma }}_{1}g_{1}(x_{1})+{\tilde{d}}_{i}+{\tilde{d}}_{1}\right)\right]+\overset{N}{\underset{i=2}{\sum }}e_{i-1}^{T}K_{i-1}^{2}e_{i-1}\;+\overset{N}{\underset{i=2}{\sum }}\left(\;{\tilde{\gamma }}_{i}{\dot{\tilde{\gamma }}}_{i}+{\tilde{d}}_{i}{\dot{\tilde{d}}}_{i}\right)+{\tilde{\gamma }}_{1}{\dot{\tilde{\gamma }}}_{1}+{\tilde{d}}_{1}{\dot{\tilde{d}}}_{1}-\overset{N}{\underset{i=1}{\sum }}\sigma_{i}{\tilde{\theta }}_{i}^{T}{\tilde{\theta }}_{i}$$

By defining the estimation error: ${\tilde{\gamma }}_{i}=\gamma_{i}-{\hat{\gamma }}_{i},$ ${\tilde{d}}_{i}=d_{i}-{\hat{d}}_{i}$. The update laws are as follows:

(29a) $${\tilde{\gamma }}_{i}{\tilde{\gamma }}_{i}+\overset{n}{\underset{j=1}{\sum }}\left|e_{i-1}^{j}\right|{\tilde{\gamma }}_{i}\;g_{i}(x_{i})=-\alpha_{i}{\tilde{\gamma }}_{i}{}^{2}\Rightarrow {\dot{\tilde{\gamma }}}_{i}=-g_{i}(x_{i})\overset{n}{\underset{j=1}{\sum }}\left|e_{i-1}^{j}\right|-\alpha_{i}{\tilde{\gamma }}_{i}\;i=2,3,\ldots ,N.\;$$

(29b) $${\tilde{\gamma }}_{1}{\dot{\tilde{\gamma }}}_{1}+\overset{N}{\underset{i=2}{\sum }}\overset{n}{\underset{j=1}{\sum }}\left|e_{i-1}^{j}\right|{\tilde{\gamma }}_{1}g_{1}(x_{1})=-\alpha_{1}{\tilde{\gamma }}_{1}{}^{2}\Rightarrow {\dot{\tilde{\gamma }}}_{1}=-g_{1}(x_{1})\overset{N}{\underset{i=1}{\sum }}\overset{n}{\underset{j=1}{\sum }}\left|e_{i-1}^{j}\right|-\alpha_{1}{\tilde{\gamma }}_{1}.$$

(29c) $${\tilde{d}}_{i}{\dot{\tilde{d}}}_{i}+\overset{N}{\underset{i=2}{\sum }}\overset{n}{\underset{j=1}{\sum }}\left|e_{i-1}^{j}\right|{\tilde{d}}_{i}=-\beta_{i}{\tilde{d}}_{i}{}^{2}\Rightarrow {\dot{\tilde{d}}}_{i}=-\overset{n}{\underset{j=1}{\sum }}\left|e_{i-1}^{j}\right|-\beta_{i}{\tilde{d}}_{i}\;i=2,3,\ldots ,N.\;$$

(29d) $${\tilde{d}}_{1}{\dot{\tilde{d}}}_{1}+\overset{N}{\underset{i=2}{\sum }}\overset{n}{\underset{j=1}{\sum }}\left|e_{i-1}^{j}\right|{\tilde{d}}_{1}=-\beta_{1}{\tilde{d}}_{1}{}^{2}\Rightarrow {\dot{\tilde{d}}}_{1}=-\overset{N}{\underset{i=1}{\sum }}\overset{n}{\underset{j=1}{\sum }}\left|e_{i-1}^{j}\right|-\beta_{1}{\tilde{d}}_{1}.$$

where $\alpha_{i}\;$and $\beta_{i}\;$are positive values. By placing the above update laws in Equation (28) we have:

(30) $$\frac{d}{dt}V\leq \overset{N}{\underset{i=2}{\sum }}\frac{1}{2}e_{i-1}^{T}K_{i-1}^{2}e_{i-1}-\overset{N}{\underset{i=1}{\sum }}(\alpha_{i}{\tilde{\gamma }}_{i}{}^{2}+\beta_{i}{\tilde{d}}_{i}{}^{2})-\overset{N}{\underset{i=1}{\sum }}\sigma_{i}{\tilde{\theta }}_{i}^{T}{\tilde{\theta }}_{i}<-\mu V\;$$

where: $\mu =\underset{i.j}{\min}\left(\alpha_{i},\beta_{i},\;\sigma_{i},k_{i-1.j}^{.}\right)>0$.

Therefore, since $K_{i-1}^{2}\;$are Hurwitz, the stability of the system is proved. Also, the convergence of synchronization errors to zero is guaranteed despite the uncertainty and disturbance. The laws for updating estimates will also be as follows:

(31a) $$\frac{d}{dt}{\hat{\gamma }}_{i}=g_{i}(x_{i})\overset{n}{\underset{j=1}{\sum }}\left|e_{i-1}^{j}\right|+\alpha_{i}{\tilde{\gamma }}_{i}\;i=2,3,\ldots ,N\;$$

(31b) $$\frac{d}{dt}{\hat{\gamma }}_{1}=g_{1}(x_{1})\overset{N}{\underset{i=1}{\sum }}\overset{n}{\underset{j=1}{\sum }}\left|e_{i-1}^{j}\right|+\alpha_{1}{\tilde{\gamma }}_{1}$$

(31c) $$\frac{d}{dt}{\hat{d}}_{i}=\overset{n}{\underset{j=1}{\sum }}\left|e_{i-1}^{j}\right|+\beta_{i}{\tilde{d}}_{i}\;i=2,3,\ldots ,N$$

(31d) $$\frac{d}{dt}{\hat{d}}_{1}=\overset{N}{\underset{i=1}{\sum }}\overset{n}{\underset{j=1}{\sum }}\left|e_{i-1}^{j}\right|+\beta_{1}{\tilde{d}}_{1}\;$$

□

**Note** **2.** *Theorems 2 and 3 are established in spite of uncertainty and disturbance, because the nature of proving them does not depend on the existence or non-existence of uncertainty and disturbance. Therefore, the problem of circular synchronization is also solved in the presence of uncertainty and disturbance.*

**Note** **3.** *If systems are time-varying:* $\theta_{i}=\theta_{i}\left(t\right)$ *Equations (9a) and (9b) are still established, so in these circumstances the Theorem 4 is also established. Therefore, as stated in note 1: circular synchronization of time-varying chaotic systems is also proven in the presence of uncertainty and disturbance.*

**Note** **4.** *The following relation can be used to make the control function continuous:*

(32) $${\bar{u}}_{i-1}{}^{j}\left(t\right)=-\left({\hat{\gamma }}_{i}g_{i}(x_{i}\right)+{\hat{\gamma }}_{1}g_{1}(x_{1})+{\hat{d}}_{i}+{\hat{d}}_{1})\cdot \tan h\left(\lambda e_{i-1}^{j}\right))\;\lambda \geq 10\;$$

**Note** **5.** *If uncertainty forms are:*$\left|\Delta f_{i}\left(x_{i}\right)\right|\leq \gamma_{i}\left|x_{i}\right|\;,i=1,2,\ldots ,N$*then in update and control laws, it should be*$\;g_{i}(x_{i})=\left|x_{i}\right|$.

**Note** **6.** *The final control function is as follows:*

$$u_{i-1}\left(t\right)=-f_{i}\left(x_{i}\right)+f_{1}\left(x_{1}\right)-H_{i}\left(x_{i}\right){\hat{\theta }}_{i}\left(t\right)+H_{1}\left(x_{1}\right){\hat{\theta }}_{1}\left(t\right)+K_{i-1}e_{i-1}-\left({\hat{\gamma }}_{i}g_{i}(x_{i}\right)\;+{\hat{\gamma }}_{1}g_{1}(x_{1})+{\hat{d}}_{i}+{\hat{d}}_{1})\cdot \tan h\left(\lambda e_{i-1}\right))$$

## 3. Application in Secure Communication Based on Chaotic Masking

In this scheme, the information signal is added to the linear composition of the master chaotic system state signals. Assuming that *S*(*t*) is the information signal carried by the master system, then the transmission message q(t) is defined as follows [64,65]:

(33) $$q\left(t\right)=S\left(t\right)+\overset{n}{\underset{i=1}{\sum }}\eta_{i}z_{i}\left(t\right)\;$$

In the given relation, $z_{i}\left(t\right)$ denotes *i*-th component of the master system and the signal q(t) is masked using the chaotic signal. This signal is transmitted from the sender to the receiver through the communication channel. Adopting the proposed controller, multi-state chaotic synchronization is conducted on one of its states. Using Equation (34), the received signal can be recovered [66]:

(34) $$\hat{S}\left(t\right)=q\left(t\right)-\overset{n}{\underset{i=1}{\sum }}\eta_{i}y_{i}\left(t\right).$$

$$\hat{S}\left(t\right)=S\left(t\right)+\overset{n}{\underset{i=1}{\sum }}\eta_{i}z_{i}\left(t\right)-\overset{n}{\underset{i=1}{\sum }}\eta_{i}y_{i}\left(t\right)$$

(35) $$=S\left(t\right)+\overset{n}{\underset{i=1}{\sum }}\eta_{i}(z_{i}\left(t\right)-y_{i}\left(t\right))=S\left(t\right)+\overset{n}{\underset{i=1}{\sum }}\eta_{i}e_{i}\left(t\right)\to S\left(t\right)\;$$

**Figure 3:**
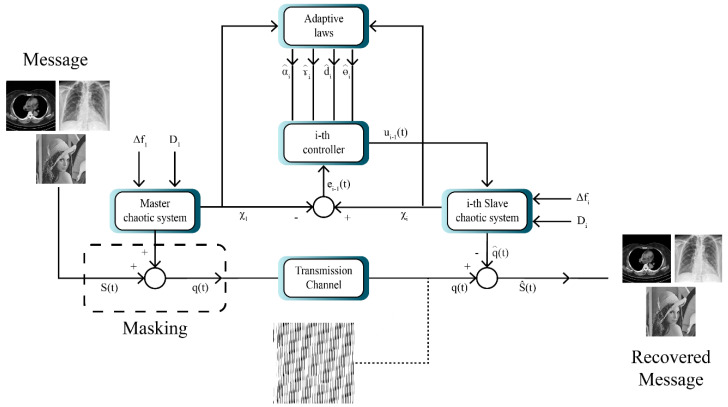
Block diagram of chaotic masking with the aid of multi-state synchronization.

The multistate system for image masking is shown in Figure 3. According to Figure 3, the model entails a master system and several slave systems. Consequently, here the message is first transmitted to the master system. In the next step, the image is encrypted and synchronized using the slave system. Finally, after synchronization in the receiver, the image is decrypted and the original messages are recovered.

Utilizing the masking method, encryption and decryption of two signals are fulfilled using the proposed multi-state synchronization method. The simulation is executed with MATLAB software. Decryption and encryption are implemented after the synchronization of the master and slave chaotic systems in the presence of disturbance and uncertainty. To that end, the given image is first converted into a signal. In the next step, this signal is added to one of the components of the master system state vector and is transmitted to the receiver through a communication channel. In the receiver part, first using the synchronization, the message signal is extracted from the chaotic signal and then converted into an image.

## 4. Implementation of the Proposed Synchronization Method on Chen Hyper-Chaotic System

A Chen hyper- chaotic system is considered as a master system and two Chen hyper chaotic systems are considered as slaves. They are defined as follows:

$$\left\{ \begin{array}{c} {\dot{x}}_{11}=\theta_{11}\left(x_{12}-x_{11}\right)+x_{14}\\ {\dot{x}}_{12}=\theta_{12}x_{11}-x_{11}x_{13}+\theta_{13}x_{12}\\ {\dot{x}}_{13}=x_{11}x_{12}-\theta_{14}x_{13}\\ {\dot{x}}_{14}=x_{12}x_{13}+\theta_{15}x_{14} \end{array}\right.$$

$$f_{1}\left(x_{1}\right)=\left[\begin{array}{c} \begin{array}{c} x_{14}\\ -x_{11}x_{13} \end{array}\\ \begin{array}{c} x_{11}x_{12}\\ x_{12}x_{13} \end{array} \end{array}\right]\;H_{1}\left(x_{1}\right)=\left[\begin{array}{ccc} \begin{array}{c} x_{12}-x_{11}\\ 0\\ \begin{array}{c} 0\\ 0 \end{array} \end{array} & \begin{array}{c} 0\\ x_{11}\\ \begin{array}{c} 0\\ 0 \end{array} \end{array} & \begin{array}{ccc} \begin{array}{c} 0\\ x_{12}\\ \begin{array}{c} 0\\ 0 \end{array} \end{array} & \begin{array}{c} 0\\ 0\\ \begin{array}{c} -x_{13}\\ 0 \end{array} \end{array} & \begin{array}{c} 0\\ 0\\ \begin{array}{c} 0\\ x_{14} \end{array} \end{array} \end{array} \end{array}\right]\;$$

Slave systems:

$$\left\{ \begin{array}{c} {\dot{x}}_{21}=\theta_{21}\left(x_{22}-x_{21}\right)+x_{24}+u_{11}\\ {\dot{x}}_{22}=\theta_{22}x_{21}-x_{21}x_{23}+\theta_{23}x_{22}+u_{12}\\ {\dot{x}}_{23}=x_{21}x_{22}-\theta_{24}x_{23}+u_{13}\\ {\dot{x}}_{24}=x_{22}x_{23}+\theta_{25}x_{24}+u_{14} \end{array}\right.$$

$$f_{2}\left(x_{2}\right)=\left[\begin{array}{c} \begin{array}{c} x_{24}\\ -x_{21}x_{23} \end{array}\\ \begin{array}{c} x_{21}x_{22}\\ x_{22}x_{23} \end{array} \end{array}\right]\;H_{2}\left(x_{2}\right)=\left[\begin{array}{ccc} \begin{array}{c} x_{22}-x_{21}\\ 0\\ \begin{array}{c} 0\\ 0 \end{array} \end{array} & \begin{array}{c} 0\\ x_{21}\\ \begin{array}{c} 0\\ 0 \end{array} \end{array} & \begin{array}{ccc} \begin{array}{c} 0\\ x_{22}\\ \begin{array}{c} 0\\ 0 \end{array} \end{array} & \begin{array}{c} 0\\ 0\\ \begin{array}{c} -x_{23}\\ 0 \end{array} \end{array} & \begin{array}{c} 0\\ 0\\ \begin{array}{c} 0\\ x_{24} \end{array} \end{array} \end{array} \end{array}\right]$$

$$\left\{ \begin{array}{c} {\dot{x}}_{31}=\theta_{31}\left(x_{32}-x_{31}\right)+x_{34}+u_{21}\\ {\dot{x}}_{32}=\theta_{32}x_{31}-x_{31}x_{33}+\theta_{33}x_{32}+u_{22}\\ {\dot{x}}_{33}=x_{31}x_{32}-\theta_{34}x_{33}+u_{23}\\ {\dot{x}}_{34}=x_{32}x_{33}+\theta_{35}x_{34}+u_{24} \end{array}\right.$$

$$f_{3}\left(x_{3}\right)=\left[\begin{array}{c} \begin{array}{c} x_{34}\\ -x_{31}x_{33} \end{array}\\ \begin{array}{c} x_{31}x_{32}\\ x_{32}x_{33} \end{array} \end{array}\right]$$

$$H_{3}\left(x_{3}\right)=\left[\begin{array}{ccc} \begin{array}{c} x_{32}-x_{31}\\ 0\\ \begin{array}{c} 0\\ 0 \end{array} \end{array} & \begin{array}{c} 0\\ x_{31}\\ \begin{array}{c} 0\\ 0 \end{array} \end{array} & \begin{array}{ccc} \begin{array}{c} 0\\ x_{32}\\ \begin{array}{c} 0\\ 0 \end{array} \end{array} & \begin{array}{c} 0\\ 0\\ \begin{array}{c} -x_{33}\\ 0 \end{array} \end{array} & \begin{array}{c} 0\\ 0\\ \begin{array}{c} 0\\ x_{34} \end{array} \end{array} \end{array} \end{array}\right]\;$$

In all examples, the parameters are set as follows:

$$K_{i-1}^{2}=Diag\left(-10,-10,-10,-10\right),\;\sigma_{i}=10,\;\alpha_{i}=20,\;\beta_{i}=20,\;i=1,2,3,4$$

***Case A*.** Multi-mode synchronization with time-varying parameters (without disturbance and uncertainty).

For parameter values, all systems are chaotic as follows:

$$\theta_{i1}=35\;\theta_{i2}=3\;\theta_{i3}=12\;\theta_{i4}=0.3\;\theta_{i5}=7,\;i=1,2,3,4,5$$

In the simulation of the basic conditions, the master system and two slave systems are selected as follows:

(*x*_11_(0), *x*_12_(0), *x*_13_(0), *x*_14_(0)) = (10, 10, 10, 10)
(*x*_21_(0), *x*_22_(0), *x*_23_(0), *x*_24_(0)) = (2, 2, 2, 2)
(*x*_31_(0), *x*_32_(0), *x*_33_(0), *x*_34_(0)) = (3, 3, 3, 3)

The initial values of the parameters are assumed to be as follows:

$${\hat{\theta }}_{1}\left(0\right)=\left[\begin{array}{c} 3\\ 3\\ \begin{array}{c} 3\\ 3 \end{array} \end{array}\right]\;{\hat{\theta }}_{2}\left(0\right)=\left[\begin{array}{c} 3\\ 3\\ \begin{array}{c} 3\\ 3 \end{array} \end{array}\right]\;{\hat{\theta }}_{3}\left(0\right)=\left[\begin{array}{c} 4\\ 4\\ \begin{array}{c} 4\\ 4 \end{array} \end{array}\right]$$

The main parameters over time are in the form of variable steps and as follows:

For a time from zero to 2 s:

*θ*_11_ = 35, _12_*θ* = 7, _21_*θ* = 35, _22_*θ* = 7, _31_*θ* = 35, _32_*θ* = 7

**Figure 4:**
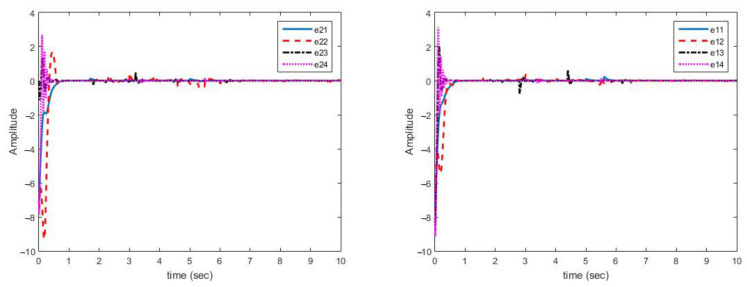
Multi-state synchronization error curves (case A).

For a time from 2 to 6 s:

*_13_*θ* = 12, _14_*θ* = 3, *_15_*θ* = 0.3, _23_*θ* = 12, _24_*θ* = 3, *θ*_25_ = 0.3, _33_*θ* = 12, _34_*θ* = 3, _35_*θ* = 0.3,

For a time from 2 to 5 s:

_11_*θ* = 33, _12_*θ* = 6, _21_*θ* = 32, _22_*θ* = 5, _31_*θ* = 34, _32_*θ* = 5,

For a time from 6 to 10 s:

_13_*θ* = 13 _14_*θ* = 1, _15_*θ* = 0.1, _23_*θ* = 12, _24_*θ* = 5, *θ*_25_ = 0.2, _33_*θ* = 10, _34_*θ* = 2, _35_*θ* = 0.2,

Figure 4 shows that synchronization errors in presence of uncertainty and disturbances are quickly converged to zero. On the other hand, parameter changes have little effect on synchronization errors and are quickly attenuated.

**Figure 5:**
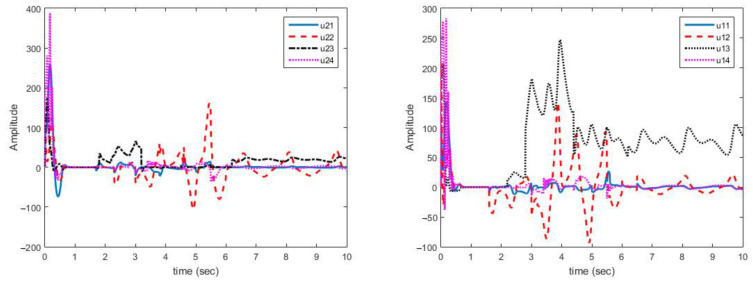
Multi-mode synchronization control effort curves (case A).

Figure 5 shows that control laws are continuous functions prevent the phenomenon of chattering.

As displayed in Figure 4, synchronization of chaotic systems as the main objective is well carried out and synchronization errors converge to zero despite changes in parameters.

As shown in Figure 5, the obtained control signals are continuous. In addition, changes in the parameters are met with a rapid reaction in the control signals to converge the synchronization errors to zero.

***Case B.*** Multi-mode synchronization with time-varying parameters despite disturbance and uncertainty. In this case, disturbance and uncertainties are applied to master and slave systems as follows:

$$\Delta f_{1}=\left[\begin{array}{c} 0.2x_{11}\sin\left(x_{11}+x_{14}\right)\\ 0.1x_{13}\sin\left(x_{11}+2x_{12}\right)\\ \begin{array}{c} 0.2\left(x_{14}+x_{12}\right)\cos\left(x_{11}-x_{13}\right)\\ x_{12}\sin\left(x_{14}+x_{12}\right) \end{array} \end{array}\right]$$

$$\Delta f_{2}=\left[\begin{array}{c} 0.5x_{22}\sin\left(x_{21}-x_{22}\right)\\ 0.2x_{24}\sin\left(x_{21}-3x_{22}\right)\\ \begin{array}{c} 0.5\left(x_{24}-x_{22}\right)\sin\left(x_{21}+2x_{23}\right)\\ 0.4\left(x_{21}-x_{22}\right)\cos\left(x_{24}-x_{22}\right) \end{array} \end{array}\right]$$

$$\Delta f_{3}=\left[\begin{array}{c} 0.3x_{32}cos\left(x_{31}+x_{34}\right)\\ 0.9x_{31}sin\left(x_{31}-x_{32}\right)\\ \begin{array}{c} 0.4x_{32}sin\left(x_{31}+x_{33}\right)\\ 0.3\left(x_{31}-x_{32}\right)sin\left(x_{34}-2x_{32}\right) \end{array} \end{array}\right]$$

$$\left|\Delta f_{i}{}^{j}\right|\leq \;\underset{j}{\max}\left|\Delta f_{i}{}^{j}\right|\leq \left|\Delta f_{i}\left(x_{i}\right)\right|\leq \gamma_{i}\left|x_{i}\right|\;\gamma_{1}=1\;\gamma_{2}=0.5\;\gamma_{3}=0.9$$

$$D_{1}=[\begin{array}{c} 0.2\sin\left(\frac{\pi }{3}t\right)\\ 0.1\sin\left(\frac{\pi }{10}t\right)\\ 0.25\sin\left(\frac{\pi }{4}t\right)\\ 0.3\sin\left(\frac{\pi }{5}t\right) \end{array}]\;D_{2}=[\begin{array}{c} 0.25\sin\left(\frac{\pi }{2}t\right)\\ 0.15\sin\left(\frac{\pi }{20}t\right)\\ 0.20\sin\left(\frac{\pi }{10}t\right)\\ 0.35\sin\left(\frac{\pi }{3}t\right) \end{array}]\;D_{3}=[\begin{array}{c} 0.3\sin\left(\frac{\pi }{30}t\right)\\ 0.2\sin\left(\frac{\pi }{20}t\right)\\ 0.15\sin\left(\frac{\pi }{10}t\right)\\ 0.1\sin\left(\frac{\pi }{25}t\right) \end{array}]$$

$$\left|D_{i}{}^{j}\left(t\right)\right|\leq \underset{j}{\max}\left|D_{i}{}^{j}\left(t\right)\right|\leq \left|D_{i}\left(t\right)\right|\;\leq d_{i}\;d_{1}=0.3\;d_{2}=0.35\;d_{3}=0.3$$

**Figure 6:**
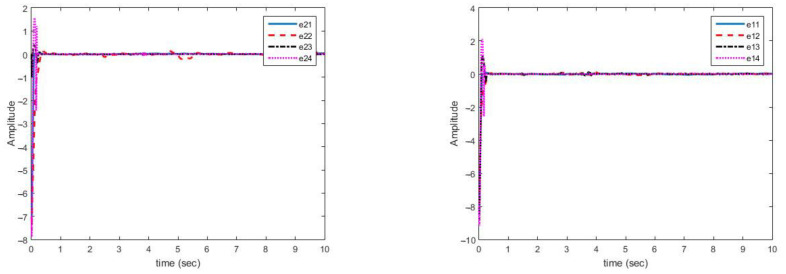
Synchronization error curves for disturbance and structural uncertainty (case B).

In this case, the change in parameters is similar to case (A). Figure 6 shows the estimation of variable parameter of the system. It can be noted that, estimating time-varying parameters in the presence of uncertainty and disturbance is correctly done.

**Figure 7:**
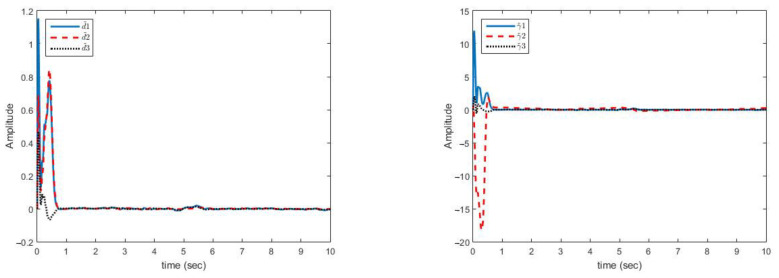
Estimation error curves of bounds related to uncertainties (**right**) and disturbances (**left**)—(case B).

Figure 6 shows the multi-mode synchronization errors despite the structural uncertainty and disturbance. We have obtained good performance as the synchronization errors are close to zero. Multi-mode synchronization errors for both master and slave systems, despite disturbance and uncertainties, have quickly reached to zero. Subsequently, change in the parameter causes small errors, which quickly converges to zero (Figure 7).

As can be seen in Figure 6, the synchronization of chaotic systems as the main aim is well done, and the synchronization errors converge to zero despite changes in parameters, presence of disturbance, and uncertainty.

***Case C***. Multi-mode synchronization, taking into account the disturbance and uncertainties bounded with the function:

For the case C$\;\left|\Delta f_{i}{}^{j}\right|\leq \;\underset{j}{\max}\left|\Delta f_{i}{}^{j}\right|\leq \left|\Delta f_{i}\left(x_{i}\right)\right|\leq \gamma_{i}g_{i}\left(x_{i}\right)$

$$\Delta f_{1}=\left[\begin{array}{c} 2x_{11}{}^{2}\cos\left(x_{11}+x_{14}\right)\\ x_{14}{}^{2}\sin\left(x_{11}+2x_{12}\right)\\ \begin{array}{c} 0.2\sin\left(x_{11}-x_{13}\right)\\ x_{13}{}^{2}\cos\left(x_{14}+x_{12}\right) \end{array} \end{array}\right].\;g_{1}\left(x_{1}\right)={\left|x_{1}\right|}^{2}+0.1\left|x_{1}\right|$$

$$\Delta f_{2}=\left[\begin{array}{c} 0.5x_{24}{}^{2}\sin\left(x_{21}-x_{22}\right)\\ 0.2x_{23}{}^{2}\cos\left(x_{21}-3x_{22}\right)\\ \begin{array}{c} 0.5\left(x_{24}{}^{2}+0.6x_{22}{}^{2}\right)\cos\left(x_{21}+2x_{23}\right)\\ 0.4\left(x_{24}{}^{2}+0.6x_{22}{}^{2}\right)\sin\left(x_{24}-x_{22}\right) \end{array} \end{array}\right].\;g_{2}\left(x_{2}\right)={\left|x_{2}\right|}^{2}\;$$

$$\Delta f_{3}=\left[\begin{array}{c} 0.3x_{31}{}^{2}cos\left(x_{31}+x_{34}\right)\\ 0.5x_{33}{}^{2}sin\left(x_{31}-x_{32}\right)\\ \begin{array}{c} 0.4{\left(x_{31}-x_{32}\right)}^{2}sin\left(x_{31}+x_{33}\right)\\ 0.3sin\left(x_{34}-2x_{32}\right) \end{array} \end{array}\right].\;g_{3}\left(x_{3}\right)={\left|x_{3}\right|}^{2}+1.2\left|x_{3}\right|$$

**Figure 8:**
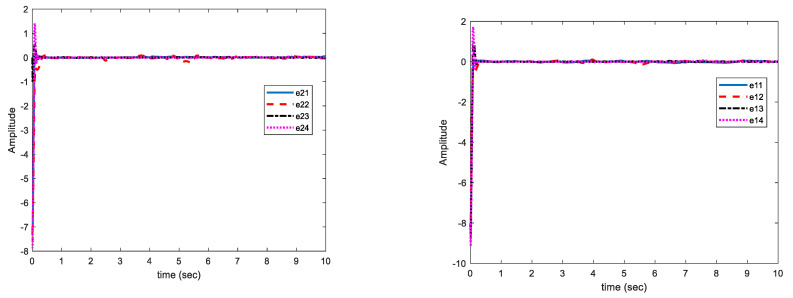
Curves of synchronization errors in case of disturbance and structural uncertainty of case (C).

In this case, the change in parameters is similar to case (A), the disturbances are similar to case (B) and uncertainties are not bounded with norm of states but bounded with a function of state norms. This increases the control problem complexity. In Figure 8, the multi-mode synchronization error in case (C) shows that despite the time-varying parameters, the functional bounded uncertainty and disturbance converge to zero.

As displayed in Figure 8, the synchronization of chaotic systems as the main objective is well fulfilled, and in spite of changes in parameters, the presence of disturbance, and uncertainty, synchronization errors are converged to zero.

**Figure 9:**
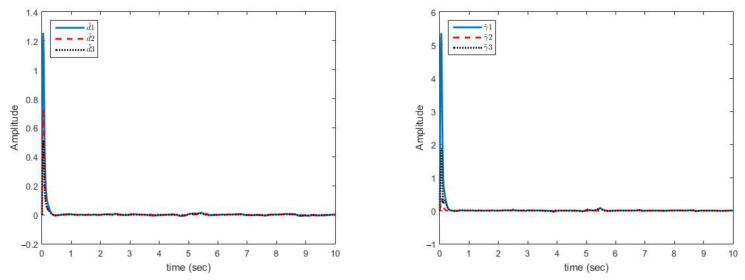
Estimation error curves for uncertainty (**right**) and disturbance (**left**) case (C).

Figure 9 shows that the estimation errors of uncertainties are more fluctuating than in case (B) but are close to zero. Estimation errors of disturbance and uncertainty bounds have reached zero with low fluctuations. The uncertainties are complex functions. The peaks appear in the figures only when the parameters have changed. However, over a short period of time, the magnitude of curves are decreased and eventually approached to zero. The proposed approach is able to reject uncertainties bounded with polynomial of state norms and unknown non-negative coefficients.

**Figure 10:**
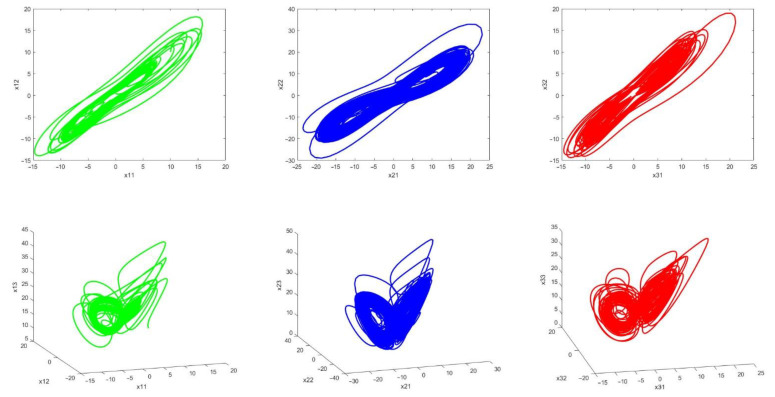
Phase curves for master chaotic system (**green**), Slave 1 (**blue**) and slave 2 (**red**) chaotic systems.

The chaotic behavior of systems is shown in Figure 10. The above figures exhibit the chaotic behavior of the master (green) and slave systems (blue and red). The presence of disturbance and uncertainty as well as changes in system parameters make the system behavior more complex.

Chaotic behavior leads to the complicatedness of secure communications method and the possibility of detecting it is so low because in chaotic systems, in addition to uncertainty and disturbance, its parameters are assumed to be variable, which promotes the security of the approach.

## 5. Statistical Metrics

### 5.1. **Histogram Analysis**

In image processing, the histogram represents the distribution of the pixel values of an image. Histogram variance is employed in image encryption. In this manner, low variance values indicate higher uniformity in encrypted images. The variance of histograms is expressed by Equation (36) [24]:

(36) $$Var\left(I\right)=\frac{1}{n^{2}}\overset{n}{\underset{i=1}{\sum }}\overset{n}{\underset{j=1}{\sum }}\frac{1}{2}{\left(I_{i}-I_{j}\right)}^{2}\;$$

According to Equation (36), I denotes the vector of histogram values, *I_i_* and *I_j_* indicate the number of pixels whose gray values are equal to *i* and *j*, respectively [24].

### 5.2. **Correlation Analysis**

Correlation distributions and correlation coefficients play a crucially significant role in the analysis of encrypted images, which are discussed in this section. The following statements are taken into account to calculate these values [24]:

(37) $$\left\{ \begin{array}{c} E\left(x\right)=\frac{1}{N}\sum_{i=1}^{N}x_{i}\\ D\left(x\right)=\frac{1}{N}\sum_{i=1}^{N}{\left(x_{i}-E\left(x\right)\right)}^{2}\\ Cov\left(x,y\right)=\frac{1}{N}\sum_{i=1}^{N}\left(x_{i}-E\left(x\right)\right)\left(y_{i}-E\left(y\right)\right)\\ \gamma_{x,y}=\frac{Cov\left(x,y\right)}{\sqrt{D\left(x\right)D\left(y\right)}} \end{array}\right.$$

### 5.3. **Differential Attack Analysis**

Pixel change rate (NPCR) and unified average changing Intensity (UACI) are two other important measurable parameters in image encryption, whose relationships of each are shown below [24]:

$$D\left(i,j\right)=\{\begin{array}{cc} 1 & I_{e1}\neq I_{e2}\\ 0 & I_{e1}=I_{e2} \end{array}$$

(38) $$NPCR=\frac{\sum_{i=1}^{M}\sum_{j=1}^{N}D\left(i,j\right)}{N\times M}\times 100\%$$

(39) $$UACI=\frac{\sum_{i=1}^{M}\sum_{j=1}^{N}\frac{\left|I_{e1}\left(i,j\right)-I_{e2}\left(i,j\right)\right|}{255}}{N\times M}\times 100\%$$

Evaluation of encrypted images by NPCR and UACI parameters is depicted in Table 1, Table 2, Table 3, Table 4 and Table 5. The results reveal that a swift change in the original image leads to a change in the ciphered image. This signifies that the proposed scheme has a high ability to resist differential attack [24].

### 5.4. **PSNR Analysis**

PSNR (peak signal to noise ratio) is the ratio of peak signal power to noise power. An encryption method achieves successful performance when the encrypted image has a low PSNR. PSNR relationships are described below [24]:

(40) $$\{\begin{array}{c} MSE=\frac{1}{M*N}\sum_{i=0}^{m-1}\sum_{j=0}^{n-1}{\left[I\left(i,j\right)-K\left(i,j\right)\right]}^{2}\\ PSNR=20*\log_{10}\left(MAX_{I}\right)-10*\log_{10}\left(MSE\right) \end{array}$$

where MSE is the mean squared error and MAXI is the maximum possible pixel value of the image [24].

### 5.5. **Information Entropy Analysis**

Information entropy is another image analysis procedure in cryptographic applications and is defined as follows [24]:

(41) $$H\left(s\right)=-\overset{2N-1}{\underset{i=0}{\sum }}P\left(s_{i}\right)\log P\left(s_{i}\right)$$

## 6. Experiment Results

The results of images encryption based on the synchronization method of the proposed chaotic systems are yielded in this section. In the following, first, the images used to perform the experiments are introduced. Then, the results of the proposed approach are expressed by application.

### 6.1. **Image Benchmarks**

**Figure 11:**
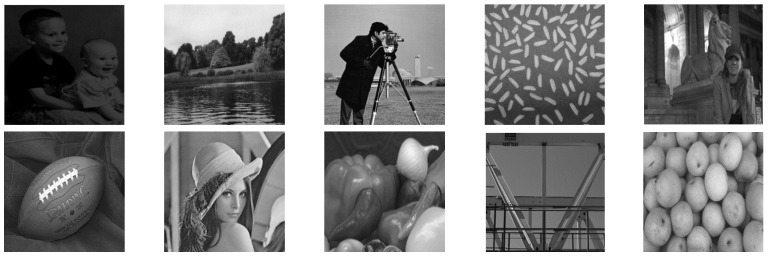
Standard benchmark images used in the proposed method.

**Figure 12:**
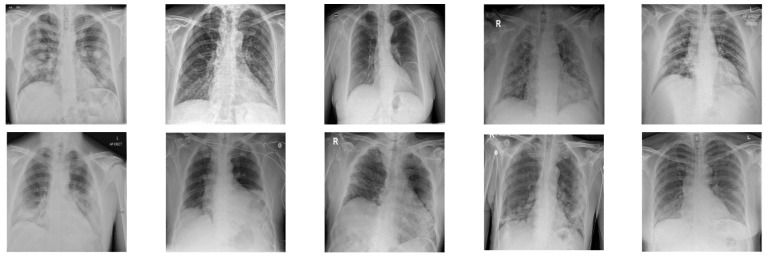
X-ray images used in the proposed method.

**Figure 13:**
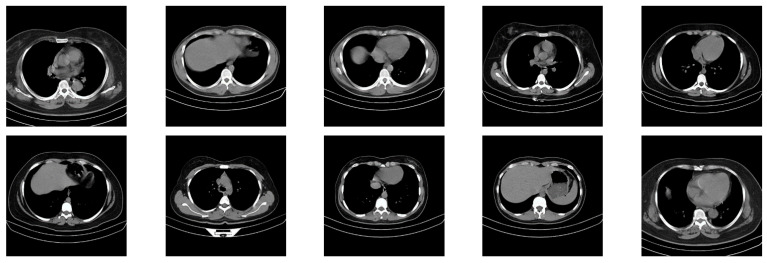
CT images used in the proposed method.

In this paper, in order to evaluate the proposed cryptographic method, multiple medical images and benchmarks have been adopted. The images tested in this section contain a variety of standard benchmark images and medical images including CT and X-ray. In Figure 11, 10 images of standard benchmarks for conducting experiments are displayed. Also, shown in Figure 12 are X-ray images of COVID-19 patients. Finally, 10 CT images of patients with COVID-19 are used for the final tests, as shown in Figure 13.

### 6.2. **Simulation**

**Figure 14:**
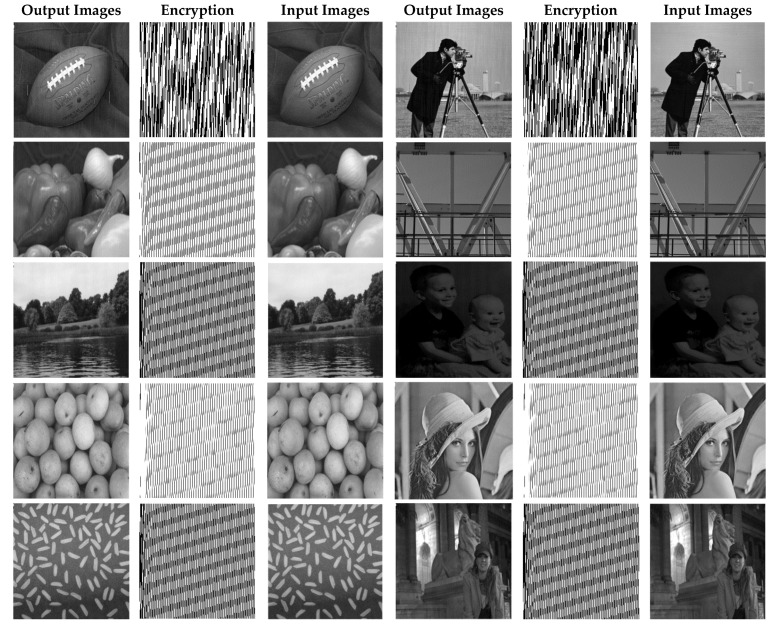
Standard benchmarks images encryption using the proposed method.

**Figure 15:**
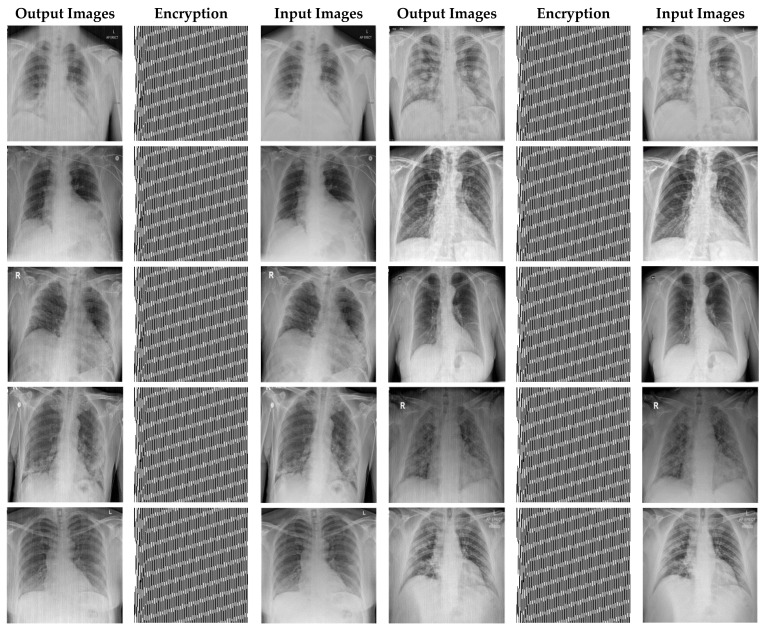
X-ray images encryption using the proposed method.

**Figure 16:**
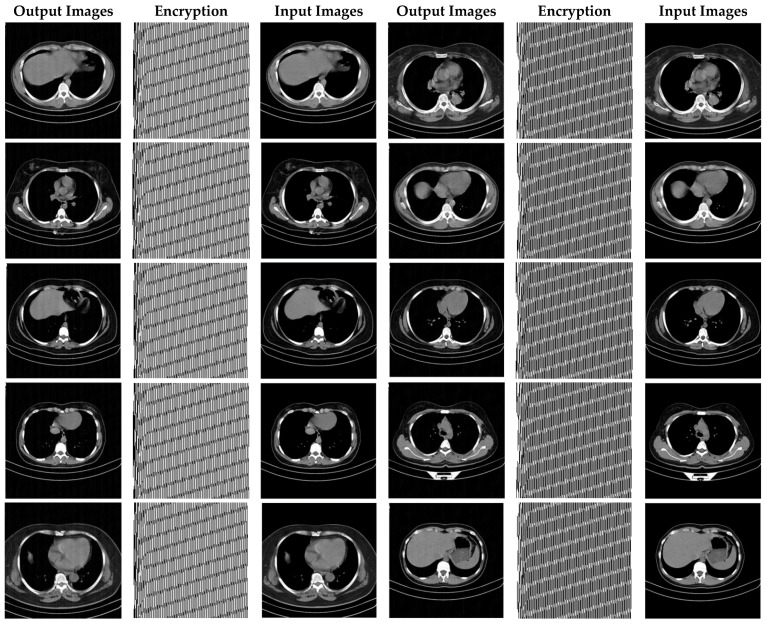
CT images encrypted using the proposed method.

In this section, the results of the proposed synchronization method of chaotic systems for various images encryption are discussed. All images utilized in this research have a size of 300 × 300 in png format. Figure 14, Figure 15 and Figure 16 show encrypted images of standard benchmark, X-ray, and CT patients with COVID-19, respectively.

The results of the proposed chaotic synchronization method for famous benchmark images encryption are shown in Figure 14. As it is manifest, the ten selected images have different contrasts, and the results displayed in Figure 14 verify the high effectiveness of the proposed techniques in the encryption of these images.

In Figure 15, X-ray images of patients with COVID-19 for cryptography are exploited. According to Figure 13, it can be seen that the synchronization method of the proposed chaotic system also achieves highly successful outcomes in X-ray images encryption.

Figure 16 demonstrates the various CT encrypted images. According to the figure, the CT images are first fed to the input of the proposed chaotic synchronization method. CT images encrypted are then generated. Finally, reconstructed CT images are represented. According to Figure 16, it can be seen that the cryptographic procedure based on synchronization of the proposed chaotic system achieves successful results.

As can be observed in Figure 16, the recommended method has also been very successful in CT images encryption of people with COVID-19. Additionally, with a conclusion, it is perceived that the cryptographic method based on synchronization of chaotic systems presented in this paper for various medical images and standard benchmarks achieves satisfactory results.

**Figure 17:**
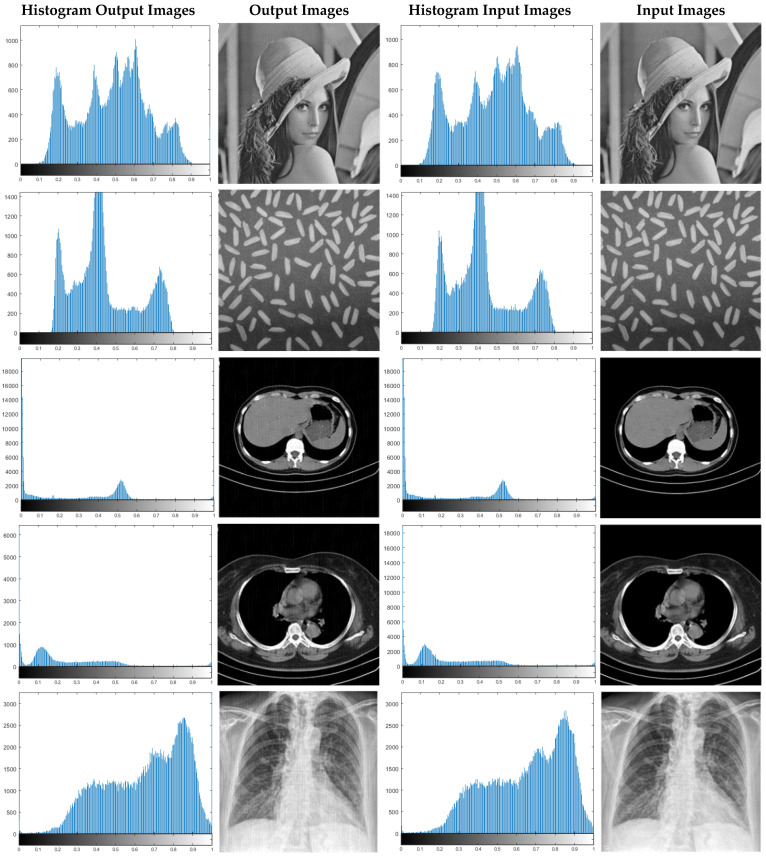
Displayed histograms for various images encrypted using the proposed method.

In Figure 17, a number of standard X-ray and CT images are randomly selected and their histogram diagrams are drawn. According to Figure 17, histograms related to input and decoded images are displayed.

In Figure 17, histogram diagrams of the number of the original input images and encrypted images are illustrated. The results show that the histograms of the input and decoded images are very close to each other. This means that the proposed synchronization method of the chaotic system has performed highly successfully in encrypting and recovering various images. For further experiments, Gaussian noises with different variances have been applied to CT images. Then, the types of statistical parameters of Section 5 are calculated for them. CT images have been corrupted by Gaussian noise with 0.001, 0.003, 0.006, and 0.009 variances, and the results of their statistical analysis are shown in Table 1, Table 2, Table 3, Table 4 and Table 5.

In Table 1, the results of the proposed methodology have been yielded on CT images without noise. As can be seen, this method has statistically satisfactory results in CT cryptography of patients with COVID-19.

In Table 2, the results of the synchronization method of chaotic systems for CT images with Gaussian noise with 0.001 variance are shown. The results in this table prove that the proposed method for encrypting CT images with Gaussian noise with 0.001 variance has been successful.

Table 3 indicates the statistical results of the proposed synchronization approach for CT images cryptography of COVID-19 patients with Gaussian noise (variance 0.003). It can be perceived that the proposed technique has been functioned successfully in encrypting these noise-corrupted CT images.

The findings of CT images encryption corrupted by Gaussian noise with 0.006 variance are given in Table 4. It is obvious that the proposed scheme for CT images for Gaussian noise with 0.006 variance compared to the previous modes has been able to report good results.

Finally, in Table 5 the results of the proposed method cryptography with CT images for Gaussian noise with 0.009 variance have been illustrated. From the results in this table, it can be seen that the proposed synchronization method is also robust to this type of Gaussian noise in CT images encryption.

## 7. Advantages and Disadvantages

The proposed method has the ability to deal with uncertainty, disturbance, and changes in system parameters. On the other hand, the resulting control functions are continuous, which prevents the chattering problems. The only problem with our proposed algorithm is the large magnitude of few control functions. In the future, we intend to develop the proposed method for synchronization of chaotic and hyper-chaotic fractional-order systems. Using an objective function as the total of control norms can reduce the magnitude of control functions.

The proposed method has strengths from various perspectives, the most important of which are discussed in the following. Ensuring the synchronization error converges to zero, which is the most mattering objective in the synchronization issue. These errors converge to zero in a short period of time, despite uncertainty and disturbance, as well as changes in the master and slave systems. Providing a continuous control function that prevents the occurrence of the chattering phenomenon. Proving the equivalence of control functions in two synchronization methods is another significant result. Finally, according to the conducted analysis, the resulting procedure has good robustness against noise, which is one of the most important outcomes of this paper.

Also, the disadvantages of the proposed method are expressed in this paragraph. In the proposed approach, there are some constraints, which incorporate: large control signal that increases the cost of synchronization. On the other hand, there is no physical factor and a significant delay in the master and slave systems. Ideal communication channel, as well as parameter changes that are considered stepwise, are other limitations of this method.

## 8. Discussion and Conclusions

Today, telemedicine is employed in a variety of medical applications. In all telemedicine systems, the issue of information security is vitally significant. To this end, various approaches for the information security of these systems have been presented so far [12]. Cryptography based on chaotic theory is one of the best approaches to protect the information in secure communications.

Encryption techniques based on chaos theory in medicine have also attracted the attention of many. In the control field, it has been proven that synchronization techniques of hyper chaotic systems to enhance information security have reported remarkably successful outcomes [54,55,56,66]. In this paper, for the first time, a novel multi-mode synchronization of a chaotic system has been utilized to encrypt medical images of CT and X-ray for COVID-19 patients. This method can be employed as an encryption section in the various telemedicine systems.

In this paper, the multi-mode synchronization of two hyper chaotic systems in two forms of multi slave, and one master system synchronization and circular synchronization are examined. Firstly, it is proved that two forms of synchronizations are equivalent and control laws are exactly equal. The adaptive control method and the definition of appropriate Lyapunov function are used for synchronization. The convergence of synchronization errors to zero is ensured despite variable parameters with time and disturbance, and uncertainty is bounded with a known function. Adaptive laws to estimate the system-time-varying parameters, disturbance, and uncertainty bounds, are also determined to ensure that the system stability is guaranteed. For preventing the phenomenon of chattering, the law of controls is established as a continuous function. To test the effectiveness of the proposed method, simulations are performed in the presence of structural uncertainty and disturbance. The results show that the proposed controller is able to perform efficiently by yielding zero errors for synchronization, estimations of disturbance, and uncertainty bounds.

After implementing the proposed multi-mode synchronization method, subsequently, medical image encryption of COVID-19 patients has been fulfilled. To verify the efficiency of the recommended method, CT images were corrupted by Gaussian noise with different variances and the statistical analysis results revealed the effectiveness of the proposed approach.

The advantages of the method involve synchronization in the presence of disturbance and uncertainty, which indicates its robustness. Using the Lyapunov function, which guarantees the convergence of all types of errors, including synchronization error and the estimation error to zero. On the other hand, the variables of the system are another great issue that makes the synchronization problem more complicated, and finally, the images are encrypted with high security, which is very difficult to detect. In Table 6, the advantages of the proposed method are compared with other researches in this field.

According to Table 6, the proposed method has more advantages than other proposals in the field of medical data encryption using chaotic methods.

For future work, the proposed method can be utilized to encrypt EEG signals of epileptic seizures [67,68], attention deficit hyperactivity disorder (ADHD) [69,70], schizophrenia [71,72], and multiple sclerosis [73]. Additionally, the technique presented in this study can be adopted to encrypt MRI [74,75], X-ray [76], mammography [77,78], and other medical data [79,80,81,82,83,84].

In the continuation of this paper in order to develop and eliminate deficiencies such as applying optimal control to synchronize with less control effort and considering the important factor of delay in master and slave systems as well as method extension for fractional-order systems in which case the complexity of the control method increases and the security of the method is enhanced. For another future works, due to the fact that medical data has uncertainty, neuro fuzzy, fuzzy type1 or fuzzy type 2 systems can be used [85,86,87,88,89,90].

## Figures and Tables

**Table 1:** Statistical metrics for CT modality without noise.

Images	Histogram		Correlation	Differential Attack		PSNR	Information Entropy
	Standard	Encrypted		NPCR (%)	UACI (%)		
Image 1	1,555,164	3,375,508	0.9970	99.611	33.461	34.623	5.5081
Image 2	1,942,777	5,880,577	0.9964	99.610	33.460	33.186	5.2665
Image 3	2,926,366	6,511,850	0.9976	99.609	33.463	34.982	4.8647
Image 4	1,969,829	5,299,018	0.9954	99.611	33.459	33.182	5.077
Image 5	2,776,087	5,805,249	0.9967	99.610	33.460	34.945	4.6828
Image 6	1,983,221	5,394,168	0.9956	99.610	33.462	33.198	5.0558
Image 7	3,108,704	6,811,427	0.9974	99.611	33.460	35.053	4.5683
Image 8	2,783,671	8,248,927	0.9958	99.610	33.460	33.483	4.5248
Image 9	2,887,118	6,433,437	0.9976	99.612	33.461	34.979	4.7115
Image 10	1,168,385	3,379,135	0.9956	99.610	33.463	32.850	5.6934

**Table 2:** Statistical metrics for CT Modality (Gaussian noise with 0.001 variance).

Images	Histogram		Correlation	Differential Attack		PSNR	Information Entropy
	Standard	Encrypted		NPCR (%)	UACI (%)		
Image 1	371,790.95	414,854.68	0.9965	99.611	33.459	34.146	6.4966
Image 2	637,672.20	775,806.22	0.9958	99.609	33.461	32.599	5.9531
Image 3	704,174.55	770,799.32	0.9969	99.610	33.462	34.284	5.8406
Image 4	655,672.19	823,316.52	0.9941	99.612	33.459	32.583	5.7021
Image 5	711,500.39	800,070.88	0.9959	99.608	33.460	34.286	5.5608
Image 6	658,916.62	837,271.82	0.9944	99.611	33.461	32.603	5.6814
Image 7	764,189.21	849,713.85	0.9966	99.612	33.463	34.304	5.5387
Image 8	910,672.69	1,120,143.86	0.9942	99.611	33.462	32.728	5.3159
Image 9	712,326.25	774,208.57	0.9969	99.613	33.458	34.276	5.6761
Image 10	377,977.28	471,233.22	0.9948	99.611	33.461	32.426	6.4675

**Table 3:** Statistical metrics for CT Modality (Gaussian noise with 0.003 variance).

Images	Histogram		Correlation	Differential Attack		PSNR	Information Entropy
	Standard	Encrypted		NPCR (%)	UACI (%)		
Image 1	239,642.29	273,559.38	0.9963	99.611	33.461	34.052	6.6729
Image 2	399,917.39	493,824.29	0.9954	99.609	33.462	32.376	6.2428
Image 3	439,060.82	492,967.11	0.9967	99.613	33.458	34.141	6.1468
Image 4	453,864.31	566,619.14	0.9937	99.610	33.460	32.416	5.9343
Image 5	490,271.52	542,562.67	0.9956	99.611	33.461	34.115	5.8208
Image 6	450,636.56	557,774.79	0.9939	99.612	33.459	32.399	5.9312
Image 7	505,486.28	558,333.49	0.9963	99.613	33.463	34.156	5.8280
Image 8	585,431.25	723,173.69	0.9936	99.609	33.462	32.481	5.6384
Image 9	456,812.35	501,600.98	0.9966	99.611	33.462	34.120	5.9823
Image 10	244,061.98	307,606.74	0.9944	99.612	33.460	32.263	6.6424

**Table 4:** Statistical metrics for CT Modality (Gaussian noise with 0.006 variance).

Images	Histogram		Correlation	Differential Attack		PSNR	Information Entropy
	Standard	Encrypted		NPCR (%)	UACI (%)		
Image 1	183,606.33	207,963.36	0.9961	99.611	33.461	33.975	6.7881
Image 2	287,175.67	347,957.53	0.9951	99.610	33.459	32.271	6.4632
Image 3	318,969.51	351,484.97	0.9965	99.608	33.458	34.027	6.3680
Image 4	353,759.37	420,480.95	0.9934	99.611	33.462	32.302	6.1234
Image 5	379,975.17	413,399.53	0.9954	99.609	33.457	34.054	6.0266
Image 6	347,921.57	414,116.53	0.9936	99.611	33.456	32.304	6.1326
Image 7	381,476.11	422,535.34	0.9962	99.612	33.461	34.097	6.0471
Image 8	435,904.80	517,108.82	0.9932	99.610	33.460	32.354	5.8833
Image 9	333,557.81	368,586.44	0.9965	99.611	33.459	34.073	6.2279
Image 10	185,554.28	227,767.28	0.9942	99.607	33.454	32.185	6.7663

**Table 5:** Statistical metrics for CT Modality (Gaussian noise with 0.009 variance).

Images	Histogram		Correlation	Differential Attack		PSNR	Information Entropy
	Standard	Encrypted		NPCR (%)	UACI (%)		
Image 1	160,406.07	181,599.02	0.9961	99.609	33.459	33.965	6.8530
Image 2	234,952.50	276,693.14	0.9950	99.611	33.461	32.237	6.6058
Image 3	262,347.29	294,388.46	0.9964	99.608	33.458	34.007	6.5026
Image 4	300,806.96	345,570.37	0.9931	99.610	33.457	32.204	6.2599
Image 5	319,370.10	347,968.94	0.9953	99.612	33.462	34.046	6.1673
Image 6	295,654.72	341,419.34	0.9934	99.607	33.457	32.213	6.2688
Image 7	320,298.48	349,954.05	0.9960	99.611	33.459	33.995	6.1951
Image 8	364,283.38	423,633.80	0.9930	99.609	33.462	32.290	6.0396
Image 9	276400.42	307161.05	0.9964	99.610	33.460	34.000	6.3806
Image 10	162565.94	191476.22	0.9940	99.612	33.461	32.139	6.8390

**Table 6:** Comparison of the proposed method with other related works.

Properties Encryption Method			Encryption Method	Data Types	Works
Disturbance	Unknown Parameter	Uncertainty			
**✕**	**✕**	**✕**	Chaos Logic Map	EEG Signals	[17]
**✕**	**✕**	**✕**	Double Chaotic Layer Encryption (DCLE)	EEG, ECG Signals	[18]
**✕**	**✕**	**✕**	Optical Chaos (Additive Chaos Masking)	EEG Signals	[19]
**✕**	**✕**	**✕**	Chaotic Modulation on the Intrinsic Mode Functions	EEG, ECG Signals	[20]
**✕**	**✕**	**✕**	Dynamic S-Boxes and Chaotic Maps	Medical Images	[21]
**✕**	**✕**	**✕**	Improvement Chaotic System	Medical Images	[22]
**✕**	**✕**	**✕**	chaotic Map + Fractional Discrete Cosine Transform (FrDCT) Coefficients	Medical Images	[23]
**✕**	**✕**	**✕**	Fourth Order Chaotic System	Medical Images	[24]
**✕**	**✕**	**✕**	Non Linear 4D Logistic Map and DNA Sequences (NL4DLM_DNA)	Medical Images	[25]
**✕**	**✕**	**✕**	Chaotic Method Based on Arnold’s Cat Map	MRI Images	[26]
**✕**	**✕**	**✕**	Latin Square + Memristive Chaotic System	Medical Images	[27]
**✕**	**✕**	**✕**	3D Chaotic Cat Map + NCA	Medical Images	[28]
**✕**	**✕**	**✕**	Multiple Chaotic Systems + MD5	Medical Images	[29]
**✕**	**✕**	**✕**	Double-Humped Logistic Map	MRI, X-ray Images	[30]
**✕**	**✕**	**✕**	Chaotic Map-based Remote Authentication Scheme	Medical Informatics	[31]
**✕**	**✕**	**✕**	Fused Coupled Chaotic Map (FCCM)	ECG Signals	[32]
**✕**	**✕**	**✕**	Rossler Dynamical Chaotic system + Sine Map	Medical Images	[33]
**✕**	**✕**	**✕**	DWT, DCT, SVD + Chaotic System	MRI Images	[34]
**✕**	**✕**	**✕**	Data Encryption Standard (DES), and Elliptic Curves Cryptography (ECC)	EEG Signals	[35]
			Chen Hyper-Chaotic System + Adaptive-Robust Multi-Mode Synchronization	Medical Images (CT, X-ray), Standard Benchmarks	Proposed Method

## Data Availability

Data sharing not applicable.
